# LILRB4 on multiple myeloma cells promotes bone lesion by p-SHP2/NF-κB/RELT signal pathway

**DOI:** 10.1186/s13046-024-03110-y

**Published:** 2024-07-01

**Authors:** Hongying Wang, Lei Wang, Huiwen Luan, Jing Xiao, Zhiling Zhao, Pengfei Yu, Mi Deng, Yifan Liu, Shuhao Ji, Junjie Ma, Yan Zhou, Jiashen Zhang, Xianhui Meng, Juan Zhang, Xinyu Zhao, Chunling Li, Fangmin Li, Dapeng Wang, Shujuan Wei, Lijun Hui, Siman Nie, Changzhu Jin, Zhiqiang An, Ningyan Zhang, Yaopeng Wang, Cheng Cheng Zhang, Zunling Li

**Affiliations:** 1 Department of Biochemistry and Molecular Biology, Shandong Tumour Immunotherapy Research Innovation Team, Binzhou Medical University, Yantai, Shandong 264003 P.R. China; 2 Department of Hematology, Yantaishan Hospital, Yantai, Shandong 264003 P.R. China; 3 Department of Biopharmaceutical, School of Pharmacy, Binzhou Medical University, Yantai, Shandong 264003 P.R. China; 4 Department of Physiology, University of Texas Southwestern Medical Center, 5323 Harry Hines Boulevard, Dallas, TX 75390 USA; 5 Peking University International Cancer Institute, Peking University, CN 38 Xueyuan Rd. Haidian Dis., Beijing, 100191 P.R. China; 6 Department of Hematology, The Affiliated Yantai Yuhuangding Hospital of Qingdao University, Yantai, Shandong 264009 P.R. China; 7 Department of Gastrointestinalstrointestinal Surgery, Yantaishan Hospital, Yantai, Shandong 264003 P.R. China; 8 Department of Biochemistry and Molecular Biology, School of Life Sciences, Shandong Agricultural University, Taian, Shandong 271018 P.R. China; 9 Department of Pathophysiology, Bengbu Medical College, Anhui, 233000 P.R. China; 10 R&D Center, Luye Pharma Group, Yantai, Shandong 264005 P.R. China; 11 Texas Therapeutics Institute, Brown Foundation Institute of Molecular Medicine, University of Texas Health Science Center, Houston, TX 77030 USA; 12 Department of Thoracic Surgery, Qingdao Hospital, University of Health and Rehabilitation Sciences (Qingdao Municipal Hospital), Qingdao, Shandong 266011 P.R. China

**Keywords:** LILRB4, Multiple myeloma, Bone lesion, RELT

## Abstract

**Background:**

Leukocyte Ig-like receptor B family 4 (LILRB4) as an immune checkpoint on myeloid cells is a potential target for tumor therapy. Extensive osteolytic bone lesion is the most characteristic feature of multiple myeloma. It is unclear whether ectopic LILRB4 on multiple myeloma regulates bone lesion.

**Methods:**

The conditioned medium (CM) from *LILRB4*-WT and -KO cells was used to analyze the effects of LILRB4 on osteoclasts and osteoblasts. Xenograft, syngeneic and patient derived xenograft models were constructed, and micro-CT, H&E staining were used to observe the bone lesion. RNA-seq, cytokine array, qPCR, the activity of luciferase, Co-IP and western blotting were used to clarify the mechanism by which LILRB4 mediated bone damage in multiple myeloma.

**Results:**

We comprehensively analyzed the expression of LILRB4 in various tumor tissue arrays, and found that LILRB4 was highly expressed in multiple myeloma samples. The patient’s imaging data showed that the higher the expression level of LILRB4, the more serious the bone lesion in patients with multiple myeloma. The conditioned medium from *LILRB4*-WT not -KO cells could significantly promote the differentiation and maturation of osteoclasts. Xenograft, syngeneic and patient derived xenograft models furtherly confirmed that LILRB4 could mediate bone lesion of multiple myeloma. Next, cytokine array was performed to identify the differentially expressed cytokines, and RELT was identified and regulated by LILRB4. The overexpression or exogenous RELT could regenerate the bone damage in *LILRB4*-KO cells *in vitro* and *in vivo*. The deletion of *LILRB4*, anti-LILRB4 alone or in combination with bortezomib could significantly delay the progression of bone lesion of multiple myeloma.

**Conclusions:**

Our findings indicated that LILRB4 promoted the bone lesion by promoting the differentiation and mature of osteoclasts through secreting RELT, and blocking LILRB4 singling pathway could inhibit the bone lesion.

**Supplementary Information:**

The online version contains supplementary material available at 10.1186/s13046-024-03110-y.

## Introduction

Multiple myeloma is incurable and characterized by abnormal clonal proliferation of malignant plasma cells, and ranks second after non-Hodgkin’s lymphoma in hematological malignancies [1]. Extensive osteolytic bone lesion is the most characteristic feature of multiple myeloma, including bone pain, osteoporosis, pathological fractures, spinal cord compression and hypercalcemia [2]. The balance between bone resorption and bone formation is maintained by osteoblasts, osteoclasts, osteocytes, bone marrow stromal cells (BMSCs) and immune cells to keep physiological bone remodeling [3], but multiple myeloma cells disrupt this balance by cell to cell dependent or independent manner to result in osteolytic bone lesions [4–6]. VLA-4 on the surface of multiple myeloma binding to the VCAM-1 in BMSCs accelerated the homing of multiple myeloma cells and modified the bone marrow microenvironment [7, 8]. Multiple myeloma cells secreted RNAKL and interleukin-6 (IL-6), endocytosed osteoprotegerin (OPG) to result in the elevated ratio of RNAKL/OPG, which promoted the differentiation and maturation of osteoclast precursors [9–13]. The drugs targeting RNAKL [14–16], syndecan-1 [17, 18], Sclerostin [19], DKK-1 [20] and RUNX2 [21] have enrolled preclinical or clinical trials. In additional, the combination of bortezomib and daratumumab [22], or thalidomide and bortezomib resulted in significantly longer progression-free survival [23]. Following autologous stem cell transplant (ASCT) significantly prolonged progression-free survival [24], and anti-BCMA CAR-T cells improved survival time in relapses or refractory multiple myeloma [25]. Although great achievements have been made in the treatment of multiple myeloma, discovery of new molecules and explore their role in bone lesions will help to develop new drugs for multiple myeloma.

As a monocytic checkpoint, leukocyte Ig-like receptor B family 4 (LILRB4) is mainly expressed on the monocytic lineage including dendritic cells, monocytes, macrophages and osteoclast [26, 27]. LILRB4 on antigen presenting cells (APC) induced CD4^+^ Th (T helper) cell anergy, and the proliferation of CD8^+^ T suppressor cells (Ts) [28–30]. Our previous study reported that apoE activated LILRB4 signaling to mediate immune escape and extramedullary infiltration of acute myeloid leukemia (AML) by cell–cell independent manner [31, 32]. LILRB4 is also expressed on tumor-infiltrating myeloid-derived suppressor cells (MDSCs) and tumor-associated macrophage to promote tumor progression [35–38]. Fibronectin or galectin-8 binding to LILRB4 could enhance the suppressive phenotype of tumor-associated myeloid cells [33, 34]. The humanized monoclonal antibody, chimeric antigen receptor T (CAR-T) cells and the antibody drug conjugates (ADC) that specifically targeted LILRB4 could significantly inhibit the progression of AML [39–41], therefore, LILRB4 has been as a potential target for tumor therapy [38]. In addition, LILRB4 has been reported to be expressed on non-small cell lung cancer to promote angiogenesis, invasion and metastasis [42], on osteoclast to inhibit differentiation and maturation of osteoclast [43], on several B lineage cells including plasma cells and plasmablasts (in particular those from lupus patients) [44], MLL-rearranged B-ALL, and on B-cell chronic lymphocytic leukemia (CLL) cells to control tumor progression [45]. Taken together, LILRB4 plays a key role in tumorigenesis and immune escape.

Concordant with its expression in certain B lineage cells, it was reported that LILRB4 is expressed in myeloma cells [40, 46]. However, it is unknown whether LILRB4 is functional in myeloma pathogenesis. In this study, we used immunohistochemistry to comprehensively analyze the expression of LILRB4 in various tumor tissue arrays, and found that LILRB4 was highly expressed in multiple myeloma samples. This result was furtherly verified in fresh multiple myeloma samples by flow cytometry, which suggested that LILRB4 was ectopic expressed on the surface of malignant plasma cells. It was reported that ectopic LILRB4 in B-cell chronic lymphocytic leukemia was involved in the infiltration of lymphoid tissues [47], controlled the progression by suppressing the Akt pathway [45], but the role of LILRB4 in multiple myeloma has not been reported. Here, we found that LILRB4 was significantly positively correlated with bone injury in multiple myeloma. LILRB4 positive rather than negative cells significantly promoted the bone lesion in xenograft, syngeneic and patient derived xenograft models. Mechanistically, fibronectin or apoE binding to LILRB4 recruited p-SHP2 to activate NF-κB signal pathway, then promoted multiple myeloma cells to secret RELT. The overexpression of RELT in *LILRB4*-KO cells significantly promoted bone lesion. The deletion of *LILRB4* or anti-LILRB4 combined with bortezomib (BTZ) could significantly delay the progression of bone lesion of multiple myeloma. Our data revealed a new function of LILRB4, elucidated a new mechanism by which multiple myeloma promotes bone damage.

## Methods

### Cells

KMS26, OPM2 and J558 were obtained from UTSW Medical Center, Raw264.7 and MC3T3-E1 were purchased from Procell Life Science&Technology Co., Ltd (Wuhan, China). All cells were confirmed by STR (Short Tandem Repeats). KMS26 and OPM2 were cultured in RPMI-1640 medium supplemented with 10% FBS and 1% penicillin–streptomycin. Raw264.7 cells were cultured in high glucose Dulbecco’s modified Eagle’s medium (DMEM) supplemented with 10% FBS and 1% penicillin–streptomycin. MC3T3-E1 (Mouse calvaria-derived preosteoblast cell line) was cultured in α-MEM medium with 10% FBS and 1% penicillin–streptomycin. 5TGM1 cell line as a valuable gift from Prof. Zhiqiang Liu (Tianjin Medical University) was cultured in RPMI-1640 medium supplemented with 10% FBS and 1% penicillin–streptomycin [48]. All cells were cultured at 37℃ in a 5% CO_2_ incubator.

### Patient sample and flow cytometry

Multiple myeloma bone marrow specimens were collected from UTSW Medical Center, Yantaishan Hospital and Yuhuangding Hospital. The criteria for inclusion and exclusion of patients are as follows: newly diagnosed patients with multiple myeloma who have not received treatment. All patients provided written informed consent, and this study was approved by the Medical Ethics Committee of Binzhou Medical University (No: 2020–17). The patient’s information was listed in Supplementary Table S1.

Bone marrow tissues were collected and subsequently analyzed by flow cytometry. PI (CAT#: P4864, Sigma), CD45-PE (CAT#: 304008, Biolegend), CD38-FITC (CAT#: 1931068, Biolegend), LILRB4-APC (CAT#: 2384215, Invitrogen), isotype-APC (CAT#: 2324760, Invitrogen) and RELT-Alexa Fluor 647 (CAT#: FAB1385R, R&D Systems) were used to label multiple myeloma cells. CD45^−^/CD38^+^ cells were malignant multiple myeloma cells [49], and LILRB4 level were detected in multiple myeloma cells.

### Mice

NOD-SCID IL2Rγ-null (NSG) mice and C57BL/6-*Rag2*^−/+^ were purchased from Shanghai Model Organisms. All animals were raised and maintained in SPF animal room of Binzhou Medical University. C57BL/6-*Rag2*^−/+^ was selfed to generate C57BL/6-*Rag2*^+/+^ and C57BL/6-*Rag2*^−/−^ homozygous mice. All experiments were approved by the Medical Ethics Committee of Binzhou Medical University (No: 2020–17), and were performed under the national standards of Institutional Animal Care and Use Committee.

### Tissue arrays

Tissue arrays of multiple myeloma (BM483b, Biomax, USA; MMP961, Wuhan Tanda Biotech CO.), prostate cancer (PR807c, Biomax, USA), liver cancer (BC03119a, Biomax, USA), melanoma (ME1004e, Biomax, USA), lung cancer (LC1201, Biomax, USA), breast cancer (BR1008a, Biomax, USA), esophagus squamous cell (ES1202, Biomax, USA), endometrium cancer (EM1021a, Biomax, USA), brain tumor (GL803c, Biomax, USA), thyroid cancer (TH801b, Biomax. USA), colorectal cancer (Hcol-Ade180Sur-07, Shanghai Outdo Biotech CO.), pancreatic cancer (Hpan-Ade120Sur-01, Shanghai Outdo Biotech CO.), head and neck cancer (HN811a, Biomax. USA) were purchased from US Biomax, lnc., Wuhan Tanda Biotech CO., Ltd or Shanghai Outdo Biotech CO., Ltd.

### Immunohistochemistry

Immunohistochemistry was performed according to our previous reports [31, 32]. In brief, all tissue arrays were deparaffinized, hydrated, antigen retrieved, blocked, incubated with primary antibodies, dehydrated and mounted. Rabbit anti-human LILRB4 antibody (Clone No: 128–3) was used to staining [39]. This antibody was a non-humanized monoclonal antibody derived from rabbit and the dilution of antibody was 1:200, and DAB exposure time was 90 secs.

### The knockout of *LILRB4* in KMS26 and OPM2 cells

*LILRB4* was knocked out by doxycycline-inducible Cas9 system according to our previous reports [31, 32]. All cells were stained by anti-LILRB4 antibody (eBioscience, ZM4.1) and LILRB4 negative cells were sorted by BD FACSAria III, and named KMS26-*LILRB4*-KO and OPM2-*LILRB4*-KO. The control cells were named KMS26-*LILRB4*-WT and OPM2-*LILRB4*-WT.

### The overexpression of *LILRB4* in 5TGM1 and J558 cells

The human *LILRB4* was overexpressed in 5TGM1 and J558 cells according to our previous reports [31, 32], and the human LILRB4 and GFP double positive cells were sorted by BD FACSAria III, and named 5TGM1-vector, 5TGM1-*LILRB4*, J558-vector and J558-*LILRB4*.

### CCK-8 cell proliferation assay

The proliferation was assessed by a CCK-8 kit (CAT#: C0038, Beyotime). Briefly, KMS26-*LILRB4*-WT, -KO and OPM2-*LILRB4*-WT, -KO cells (5 × 10^3^cells/well) were seeded in a 96-well plate. After 0, 12, 24, 48, and 72 h, 10μL CCK-8 solution was added and incubated for 1 h at 37°C. The relative optical density at 450 nm was detected by a spectrophotometer (SpectraMax M2, Molecular Devices).

### The conditioned medium treated osteoclasts and osteoblasts

KMS26-*LILRB4*-WT, -KO and OPM2-*LILRB4*-WT, -KO cells (1 × 10^6^/ml) were suspended in RPMI-1640 medium and seeded into 6-well plate where the recombinant human apoE (Cat#:C102, Novoprotein) were coated at 4℃ overnight. After 48 h, the conditioned medium (CM) were collected, aliquoted and stored in -80℃。

MC3T3-E1 cells (2 × 10^4^/ml) were seeded into 24-well plates. After 24 h, MC3T3-E1 cells were induced by 50 µg/ml ascorbic acid (Cat#: A92902, Sigma-Aldrich), 10 mM sodium β-glycerophosphate (Cat#: G4922, Sigma-Aldrich), 100 nmol/L dexamethasone (Cat#: D4902, Sigma-Aldrich) and 10% conditioned medium (CM) from *LILRB4*-WT or -KO cells. Cell morphology was observed every 24 hours, and alkaline phosphatase activity assay was detected on day 7, and alizarin red staining was performed on day 14.

The mouse macrophage cell line Raw264.7 was seeded into 96-well plates (1 × 10^4^/each well). After 24h, the cells were treated with 100 ng/ml RNAKL (Cat#: R0525, Sigma-Alrdich), 25 ng/ml M-CSF (Cat#: M9170, Sigma-Alrdich) and 10% CM from *LILRB4*-WT or -KO cells. After 4 days, cells were stained for TRAP using a TRAP/ALP stain kit (Cat#: 294–67,001, Wako), and the multinucleated cells (the number of nuclei was more than or equal to 3) were counted under the microscope.

For primary mouse cell co-culture, bone marrow derived mononuclear cells from C57BL/6 mice at the age of 6–8 weeks were cultured in 96-well plates in α-MEM supplemented with 10% FBS, 25 ng/ml M-CSF, and 100 ng/ml RANKL in the presence of J558-vector or -*LILRB4* conditioned medium for 6 days. The cells were fixed in formalin and stained for TRAP using a TRAP staining kit according to the instructions. TRAP^+^ cells containing 3 or more nuclei were counted as osteoclasts.

### The overexpression of *RELT* in KMS26-*LILRB4*-KO cells

The RNA was extracted from KMS26-*LILRB4*-WT cells using RNAiso Plus reagent (CAT#:9108, TAKARA) and reverse-transcribed using PrimeScript™ II 1st Strand cDNA Synthesis Kit (CAT#:6210A, TAKARA) according to the manufacturer’s manual. The specific primer of *RELT* was as follows, and underline indicated the in-fusion sequence.

F: ATTTCCGGTGAATTCATGAAGCCAAGTCTGCTGTGCCGGC;

R: CGCTCTAGAACTAGTTCAGATGACCAGGTTGCTCTCACTT

The cDNA of *RELT* was cloned into the lentivirus vector by in-fusion, and correct by sequencing. The human *RELT* expression plasmids were overexpressed in KMS26-*LILRB4*-KO cells. The RELT positive cells named KMS26-*LILRB4*-KO-*RELT* were sorted by BD FACSAria III, and KMS26-*LILRB4*-KO-vector cell as control.

### Cytokine antibody arrays

Cytokine Antibody Arrays (RayBio ® Human Angiogenesis Antibody Array G-Series 1000, CAT #: AAH-ANG-G1000-4, RayBiotech, Inc.) were used to detect the levels of cytokine in the conditioned medium as described in manufacturer’s protocol. In a short, 100 μl 1 × blocking buffer was added into each well and incubated at room temperature for 30 min to block slides, then aspirated from each well. Next, 100 μl sample was added and incubated at room temperature for 2 hours. After washing, 70 μl 1 × biotin-conjugated anti-Cytokines were added to each well, and incubated at room temperature for 2 hours with gentle rocking. Washing steps were repeated, followed by addition of 70 μl of 1 × Streptavidin-Fluor to each sub-array to incubate at room temperature for 2 hours in dark room with gentle rocking. Streptavidin-Fluor reagent were aspirated from each well carefully before washing. The signals of cytokine array were scanned with a microarray scanner (InnoScan 300, Innopsys, France) at the appropriate wavelength (532 nm) for Cy3, and fluorescence intensity data were analyzed using Raybiotech Software (RayBiotech, Peachtree Corners, Georgia, USA).

### RNA-sequencing

2 × 10^6^ KMS26-*LILRB4*-WT, -KO and OPM2-*LILRB4*-WT, -KO cells were cultured and collected, and all cells (3 replicates for each cell) were mailed to BGI in dry ice. The RNA extraction, quality control and sequencing were completed by GBI, and all data were analyzed in BGI Dr. Tom system (https://biosys.bgi.com/).

### Real time PCR

Total RNA was extracted from cells using RNAiso Plus reagent (CAT#:9108, TAKARA) and reverse-transcribed using PrimeScript™ II 1st Strand cDNA Synthesis Kit (CAT #:6210A, TAKARA) according to the manufacturer’s manual. 100 ng cDNA products were used for subsequent PCR analysis through ChamQ Universal SYBR qPCR Master Mix (CAT#: Q-711, Vazyme) on QuantStudio™ Design & Analysis SE Software (Thermo Fisher Scientific). The specific primer for target genes were as follows.

VEGF

F: 5’-GTCCGGACTCGACCTCTCG-3’

R: 5’-TCTACACTGGACACAGACCG-3’,

RANKL

F: 5’-CAACATATCGTTGGATCACAGCA-3’

R: 5’-GACAGACTCACTTTATGGGAACC-3’,

P53

F: 5’-CAGCACATGACGGAGGTTGT -3’

R: 5’-TCATCCAAATACTCCACACGC -3’,

RELT

F: 5’-GTTCCATGTCAACCATGTTCCT-3’

R: 5’-AGGCAGAAGACAGGGACGAT-3’,

TRPC1

F: 5’-AGGATAGCCTCCGGCATTC-3’

R: 5’-TTCCACCTCCACAAGACTTAGT-3’

GAPDH

F: 5’- ACAACTTTGGTATCGTGGAAGG-3’

R: 5’-GCCATCACGCCACAGTTTC-3’

### Co-immunoprecipitation

KMS26-*LILRB4*-WT and -KO cells were cultured, collected and washed twice with ice PBS (pH 7.4). Then, the cells were re-suspended by IP lysis buffer with protease inhibitors cocktail (Cat#: 04693116001, Roche) and incubated on ice for 30 mins. The supernatant was collected by centrifugation (4°C, 14,000 g, 10 mins). The cell lysate (600 μg) was divided into two parts and incubated with IgG or 128–3 [39] (anti-LILRB4) overnight at 4°C on a shaker, respectively. Next, Protein G Magnetic Beads were added and incubated for 2 h at 4°C on a shaker. Protein G Magnetic beads-Ab-Ag complex was washed 3 times, and heated at 95°C for 5 min in 1 × loading buffer. The samples were separated by SDS-PAGE, and anti-phospho-SHP2 (Y580, Cat#:5431, Cell signaling technology) was incubated overnight.

### ELISA

The levels of RELT in patient serum and conditioned medium (CM) were measured using a RELT ELISA Kit (CAT#: SEK10530, Sino biological). Briefly, diluted captured antibodies were coated to a 96 well plate and incubated overnight at 4℃. Standards and samples were added into the wells, and RELT bound to the immobilized antibody. The wells were washed and a horseradish peroxidase conjugated rabbit anti-human RELT monoclonal antibody was added. The wells were washed and TMB substrate solution was loaded. To end the enzyme reaction, the stop solution was added and absorbances of the microwell were read at 450 nm.

### Western blotting

2 × 10^6^ KMS26-*LILRB4*-WT or -KO cells were cultured, and a nuclear and cytoplasmic extraction kit (CAT#: 788330, Thermo Fisher Scientific) was used to extract the nuclear and cytoplasmic proteins. Primary antibodies including anti-phospho-SHP2 (Y580, Cat#:5431, CST, 1:1000), anti-SHP2 (Cat#:3397, CST, 1:1000), anti-phospho-p65 (Ser536, Cat#:3033, CST, 1:1000), anti-p65 (Cat#:8242, CST, 1:1000) and anti-RELT (Cat#:146,955, Absin, 1:1000) were incubated overnight at 4℃.

The conditioned medium (CM) from KMS26-*LILRB4*-KO-vector or -*RELT* cells was collected. Raw264.7 cells were treated with 10% CM. After 4 days, cells were lysed and RELT-associated signaling proteins were detected by western blotting. The primary antibody was as follows, anti-phospho-p65 (Ser536, Cat#:3033, CST, 1:1000), anti -p65 (Cat#:8242, CST, 1:1000), anti-NFATC1 (Cat#:8032, CST, 1:1000), anti-c-fos (Cat#:2250, CST, 1:1000), anti-phospho-c-fos (Ser32, Cat#:5348, CST, 1:1000), anti-phospho-Akt (Ser473, Cat#:4060, CST, 1:1000), anti-Akt (Cat#:4691, CST, 1:1000), anti-phospho-MEK1/2 (Ser217/221, Cat#:9154, CST, 1:1000), anti-MEK1/2 (Cat#:9122, CST, 1:1000), anti-ERK1/2 (Cat#:YT1625, Immunoway biotechnology, 1:1000), anti-phospho-ERK1/2 (T204, Cat#:YP0101, Immunoway Biotechnology, 1:1000). The quantification of the bands were performed by Image J.

### Luciferase reporter assay

The core promoter of *RELT* was amplified from the genomic DNA of KMS26 cells, and cloned into pGL3-basic luciferase reporter vector by in-fusion, and was correct by sequencing. The primer was as follows, and underline indicated the in-fusion sequence.

F: 5’-TAGCCCGGGCTCGAGTTTAGAACCCCACCCCCAGCCAT-3’

R: 5’-CGGAATGCCAAGGCTTACACGGGGAGGCAGGGGGCGCCA-3’

The expression vector of *LILRB4* was conserved by our lab [31, 32], and the coding sequence (CDS) of *p65* was amplified and cloned into pcDNA3.1( +) by in-fusion. The specific primer targeting *p65* was as follows and the underline indicated the in-fusion sequence. The *p65* construct was correct by sequencing.

F: 5’-TTTAAACTTAAGCTTATGGACGAACTGTTC-3’

R: 5’-ATATCTGCAGAATTCTTAGGAGCTGATCTGACTCA-3’

Next, 0.5 μg of luciferase reporter vector and 0.02 μg of the pRL-TK renilla reniformis lucidferase as a normalizing control were co-transfected into OPM2-*LILRB4*-WT and -KO cells. 0.5 μg of luciferase reporter vector and 0.3 μg of LILRB4 or p65 expression constructs with 0.02 μg of the pRL-TK renilla reniformis lucidferase were co-transfected into OPM2-*LILRB4*-KO cells. The Dual Luciferase Assay System (CAT#: E2920, Promega) was used to analyze the luciferase activity.

### Multiple myeloma mouse model by tail vein

A total of 1 × 10^6^ KMS26- and OPM2-*LILRB4*-WT, -KO cells were collected and re-suspended in 200 µl ice PBS (pH = 7.4), and the cells were injected into NSG mice by tail vein. For the KMS26 mice model, tumor progression was monitored by bioluminescence imaging (IVIS Lumina, PerkinElmer). After 5 weeks, the mice were sacrificed under anesthesia. The mice were raised until death in order to plot the survival curves in OPM2 mice model. Flow cytometry was used to detect the GFP^+^ cell percentage in peripheral blood. The skull and tibia were stripped and fixed in formalin, and the bone lesion was scanned by micro-CT (Quantum GX, PerkinElmer).

### 5TGM1 multiple myeloma mouse model

A total of 1 × 10^6^ 5TGM1-vector and -*LILRB4* cells were cultured, collected and re-suspended in 200 µl ice PBS (pH = 7.4), then were injected into C57BL/6-*Rag2*^−/−^ homozygous mice by tail vein. Bioluminescence imaging (IVIS Lumina, PerkinElmer) was used to monitor the progression of multiple myeloma. After 35 days, the mice were sacrificed under anesthesia, and micro-CT was used to scan bone lesion.

### Multiple myeloma mouse model by intratibial injection

KMS26-*LILRB4*-WT, KMS26-*LILRB4*-KO-vector and -*RELT* cells were prepared, and NSG mice (4–6 weeks, female) were anesthetized by isoflurane. The tibial plateau was shaved and sterilized with 75% medical ethanol. At 5 mm below the knee joint, 3 mm incision fully exposed the tibia plateau, and 1 ml syringe was used to make a hole. The cells (1 × 10^6^ in 10 µl micro-injector) were slowly injected into bone cavity, and the incision was sterilized. After 7 days, micro-CT was used to scan bone lesion.

### PDX model of multiple myeloma

The bone marrow of newly diagnosed patients with multiple myeloma was provided in sterile anticoagulant tube by Yantaishan and Yuhuangding Hospital. CD45^−^CD38^+^LILRB4^−^ and CD45^−^CD38^+^LILRB4^+^ cells were sorted by BD FACSAria III. The same amount of cells were injected into NSG mice (4–6 weeks, female) by intratibial injection. When mice died naturally, the bone lesion was observed by micro-CT.

### Multiple myeloma mouse model was treated by BTZ

A total of 1 × 10^6^ KMS26-*LILRB4*-WT and -KO cells were injected into NSG mice (4–6 weeks, female) by tail vein. After 7 days, BTZ (1.0 mg/kg) or PBS were injected into NSG mice by intraperitoneal injection and twice each week. The mice were sacrificed under anesthesia on the 33^rd^ day, and the bone lesion was evaluated by micro-CT.

### Anti-LILRB4 alone or in combination with BTZ was used to treat multiple myeloma mouse model

Anti-LILRB4 was produced according to our previous report [39]. A total of 1 × 10^6^ KMS26-*LILRB4*-WT cells were injected into NSG mice by tail vein. Isotype (CAT#: A2051, Selleck) (200 μg/each mouse), anti-LILRB4 (200 μg/each mouse) or BTZ (1.0 mg/kg) were used to treat the mouse model every 3 days from the 2^nd^ day. After 40 days, the mice were sacrificed, and bone was scanned by micro-CT.

### Histology

The tibia was fixed with paraformaldehyde, decalcified with 10% EDTA for two weeks, embedded in paraffin, and sectioned along the mid-sagittal plane in 4.5 mm-thick sections. Hematoxylin and eosin (H&E) staining was performed to observe bone morphology.

### Micro-CT analysis

The formalin on the bone sample was cleaned, and the bone was scanned using a small animal *in vivo* micro-CT imaging system (Quantum GX, PerkinElmer) with 4.5 μm voxel size for 14 min, X-ray source set to 80000 mGy, 90 kV and 88μA. To observe cortical bone lesions, the entire femur image was imported into the software for 3D reconstruction using the same threshold. For trabecular microstructure, 100 slices, corresponding to a 1 mm region underneath the growth plate, were obtained with 10 μm spatial resolution.

### Statistical analysis

The relationship between the LILRB4 expression and bone lesion was analyzed by the linear regression in GraphPad Prism 10.0 software. One-way ANOVA or t test was used to analyses the differences among the different groups.

## Results

### LILRB4 was expressed in multiple myeloma

**Fig. 1:**
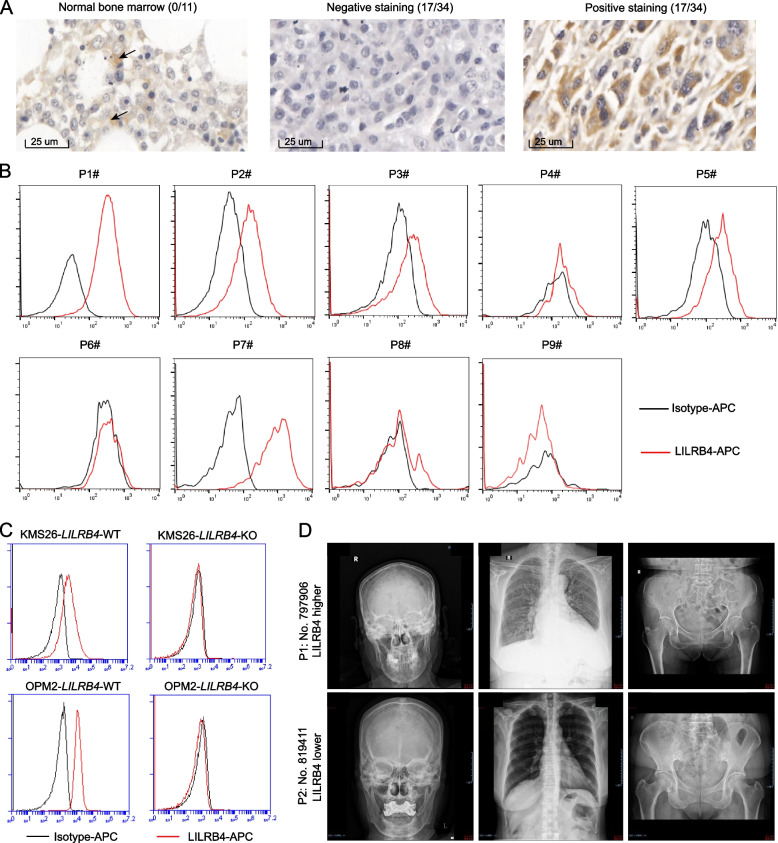
LILRB4 was expressed in multiple myeloma. LILRB4 was detected by IHC in tissue array of multiple myeloma (the arrow indicated the LILRB4 expression in normal bone marrow, *n* = 34, **A**), by flow cytometry in fresh patient samples (*n* = 9, **B**). *LILRB4* was knocked out by CRISPR-Cas9 in KMS26 and OPM2 cell lines (**C**). The imaging data showed the relationship between LILRB4 level and bone damage (*n* = 16, **D**)

LILRB4, a member of the leukocyte immunoglobulin-like receptor B family, was reported to be expressed in hematological and solid tumors [31, 32, 42, 50, 51]. In order to clarify the expression pattern of LILRB4 in tumor cells, we queried LILRB4 expression in The Cancer Genome Atlas (TCGA) and The Human Protein Atlas database. TCGA showed that the mRNA of *LILRB4* was significantly upregulated in breast invasive carcinoma (BRCA), cholangiocarcinoma (CHOL), esophageal carcinoma (ESCA), head and neck squamous cell carcinoma (HNSC), kidney chromophobe (KICH), kidney renal clear cell carcinoma (KIRC), kidney renal papillary cell carcinoma (KIRP), prostate adenocarcinoma (PRAD), skin cutaneous melanoma (SKCM), stomach adenocarcinoma (STAD) and uterine corpus endometrial carcinoma (UCEC) compared to normal tissue (https://cistrome.shinyapps.io/timer/). The Human Protein Atlas also showed that LILRB4 could be detected in colon cancer samples (https://www.proteinatlas.org/ENSG00000186818-LILRB4/pathology). However, the expression pattern of LILRB4 in most tumor cells has not been reported. We detected the expression of LILRB4 in all kinds of tumor tissue arrays by immunohistochemistry (IHC). LILRB4 was expressed in 50% multiple myeloma (17/34) (Fig. 1A), 8% prostate cancer (4/50) (Supplementary Fig. S1A), 4.5% hepatocellular carcinoma (5/110) (Supplementary Fig. S1B) and 11.9% melanoma (10/84) (Supplementary Fig. S1C). By contrast, LILRB4 was not detected in lung cancer (0/100), breast cancer (0/90), esophageal cancer (0/90), endometrial cancer (0/100), brain tumor (0/45), thyroid cancer (0/40), colorectal cancer (0/90), pancreatic cancer (0/80) and head and neck cancer (0/19) (Supplementary Fig. S1D-L). Our results suggested that LILRB4 was highly expressed in multiple myeloma cells.

Next, we collected 9 fresh specimens of multiple myeloma from UT Southwestern Medical Center and detected LILRB4 level by flow cytometry. CD45^−^/CD38^+^ cells were malignant multiple myeloma cells [49]. LILRB4 was detected in 55.56% multiple myeloma samples (5/9, Fig. 1B and Supplementary Fig. S2A). Moreover, LILRB4 was also expressed in multiple myeloma cell lines KMS26 and OPM2. *LILRB4* was knocked out by CRISPR-cas9 technology in KMS26 and OPM2 cells (Fig. 1C). Meanwhile, the knockout of *LILRB4* did not alter the proliferation of KMS26 and OPM2 cells (Supplementary Fig. S2B, C), indicating that LILRB4 did not regulate the proliferation of multiple myeloma.

Extensive osteolytic bone lesion is the most characteristic feature of multiple myeloma. Although LILRB4 expressed on the surface of osteoclast negatively regulated osteoclastogenesis [43, 52], the role of LILRB4 on bone lesion of multiple myeloma has not been reported. We collected bone marrow specimen and imaging data of 16 newly diagnosed patients with multiple myeloma, and found that the higher the expression level of LILRB4, the more serious the bone lesion (Fig. 1D and Supplementary Fig. S2D), suggesting that there was a certain trend and LILRB4 may be involved in bone injury in multiple myeloma.

### The conditioned medium from *LILRB4*-WT not -KO cells significantly promoted osteoclastogenesis

Osteoclast is the only cell that can promote bone resorption [53], and multiple myeloma cells promotes the differentiation and maturation of osteoclasts [54]. It has not been reported whether LILRB4 on multiple myeloma cells is involved in osteoclastogenesis. The conditioned medium (CM) from *LILRB4*-WT and -KO cells was used to treat the mouse macrophage cell line Raw264.7. Compared to the PBS group, RANKL could induce the osteoclastogenesis (more than 3 nuclei, Supplementary Fig. S3A, B). The CM from KMS26 or OPM2 could significantly induce the differentiation and maturation of osteoclast, and the numbers of osteoclasts in *LILRB4*-WT group were significantly higher than that in *LILRB4*-KO group (Supplementary Fig. S3A, B). Next, human *LILRB4* was overexpressed in mouse myeloma cell line J558, and the CM was used to treat the primary mouse bone marrow mononuclear cells. The CM from J558-*LILRB4* cells could significantly induce osteoclastogenesis (Supplementary Fig. S3C, D) when compared with the CM from J558-vector cells, suggesting that LILRB4 on multiple myeloma cells could induce osteoclastogenesis.

To determine the effect of myeloma-expressing LILRB4 on osteoblasts, we employed the CM of *LILRB4*-WT or -KO cells to treat MC3T3-E1 cell line (mouse embryonic calvarial fibroblasts). Alizarin red and ALP staining were used to characterize the maturation of osteoblasts. After 14 days of CM treatment, the cellular boundary was unclear, the morphology disappeared, and the apoptosis was obvious, indicating the CM from multiple myeloma, whether LILRB4 was knocked out or not, could induce the apoptosis of osteoblasts (Supplementary Fig. S4A-D). There was no significant difference between these two groups by alizarin red and ALP staining (Supplementary Fig. S4A-D), indicating the knockout of *LILRB4* did not improve the osteoblasts apoptosis mediated by multiple myeloma, and also did not promote the differentiation and maturation of osteoblasts.

These results suggested that LILRB4 on multiple myeloma promoted the osteoclastogenesis, and had no effect on the differentiation and maturation of osteoblasts.

### LILRB4 promoted osteolytic lesions *invivo*

**Fig. 2:**
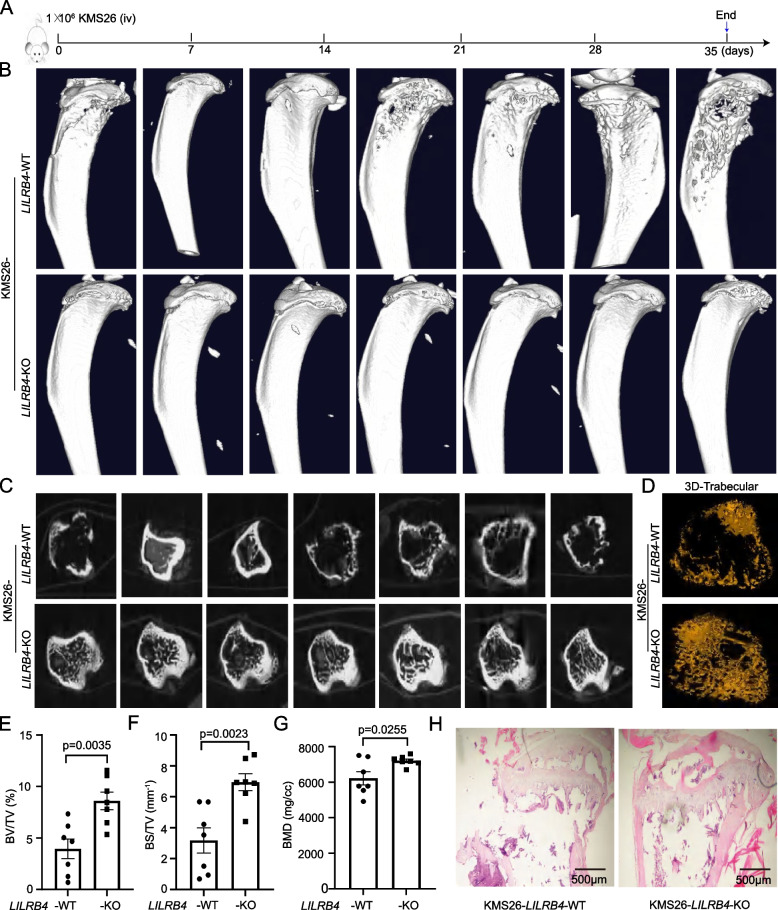
LILRB4 promoted bone injury in KMS26 xenograft model. The schematic of KMS26 xenograft model (**A**). Micro-CT was used to scan the bone lesion after 35 days. 3D reconstruction of tibia (**B**), transverse plane of trabecular (**C**) and 3D-trabecular (**D**) showed the tibial morphology and the structure of the trabecular. Trabecular bone volume/tissue volume (BV/TV, **E**), trabecular bone surface/tissue volume (BS/TV, **F**) and bone mineral density (BMD, **G**) were analyzed by micro-CT (*n* = 14). Representative bone lesions were observed in H&E staining (**H**)

Next, we used different animal models [55] to study whether LILRB4 in multiple myeloma can mediate osteolytic lesions. KMS26-*LILRB4*-WT and -KO cells were injected into NSG mice by tail vein (Fig. 2A). After 35 days, the mice showed detectable somatic and nervous system symptoms, such as paraplegia of the hind limbs, fixed lap running, and all mice were sacrificed under anesthesia. Bioluminescence imaging and the numbers of tumor in abdominal cavity showed no significant difference in proliferation of KMS26-*LILRB4*-WT and-KO cells *in vivo* (Supplementary Fig. S5A-D). The 3D reconstruction of tibia showed that five of the seven mice had obvious bone fractures and bone lesions in KMS26-*LILRB4*-WT group, however, the tibia of all mice in KMS26-*LILRB4*-KO group were intact, and there was no visible bone damage (Fig. 2B). Trabecular bones were almost completely destroyed, bone cortex was discontinuum, and 3D-trabecular had obvious defects in *LILRB4*-WT not -KO group (Fig. 2C, D). Micro-CT analysis of tibial trabecular bone volume / tissue volume (BV/TV), trabecular bone surface / tissue volume (BS/TV) and bone mineral density (BMD) were significantly lower in *LILRB4*-WT group than that in *LILRB4*-KO group (Fig. 2E-G). Histological analysis demonstrated that cortical and trabecular bone had obvious lesions in KMS26-*LILRB4*-WT not -KO group (Fig. 2H). In OPM2 xenograft models, 4 out of 5 mice (80%) showed significant cracking in their cranial sutures in *LILRB4*-WT group, but only 2 mice (40%) showed crack in *LILRB4*-KO group (Supplementary Fig. S6A), and the knockout of *LILRB4* could significantly prolong the survival time of mice (Supplementary Fig. S6B). LILRB4 significantly promoted bone lesion, and trabecular destruction was more serious (Supplementary Fig. S6C, D). BV/TV, BS/TV and BMD also showed that *LILRB4*-WT not -KO group had stronger bone destruction (Supplementary Fig. S6E-G). There is no significant difference in the proportion of tumor cells in peripheral blood (Supplementary Fig. S6H). The above results showed that LILRB4 did not promote the proliferation of multiple myeloma, and the bone lesion mediated by LILRB4 was not caused by the proliferation of multiple myeloma cells.

**Fig. 3:**
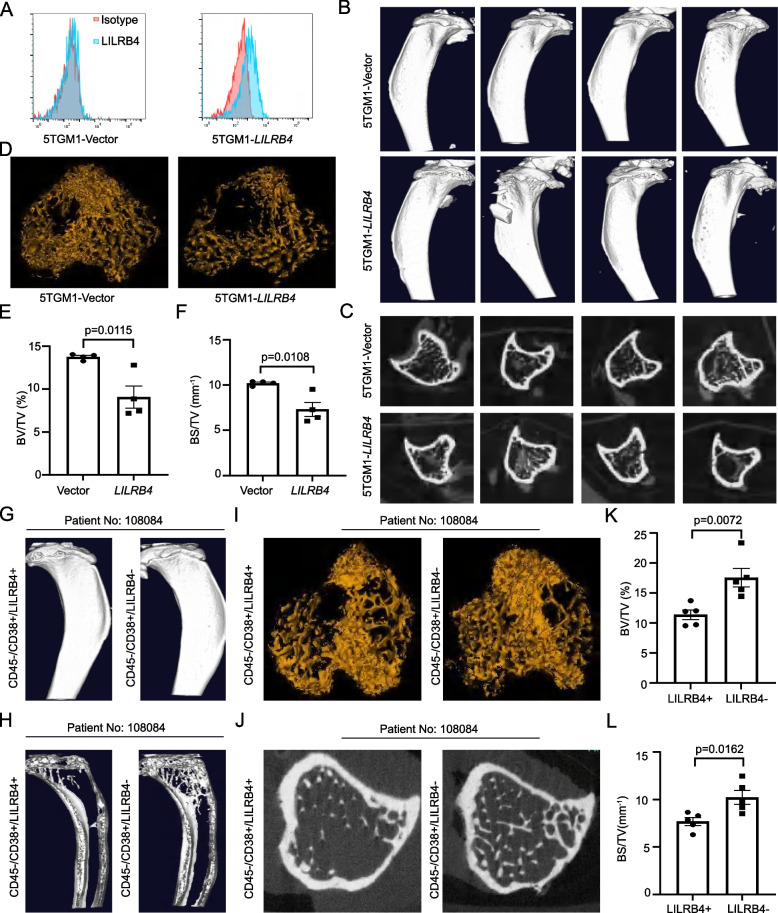
The syngeneic and PDX model showed that LILRB4 mediated the bone lesion of multiple myeloma. Human *LILRB4* was overexpressed in 5TGM1 cells (**A**), and 5TGM1-vector and -*LILRB4* cells were injected into C57BL/6-*Rag2*^−/−^ mice. The 3D structure of tibia (**B**), transverse plane of trabecular (**C**), 3D trabecular (**D**), BV/TV (**E**) and BS/TV (**F**) were analyzed by micro-CT (*n* = 8). CD45^−^/CD38^+^/LILRB4^+^ and CD45^−^/CD38^+^/LILRB4.^−^ cells were sorted and injected into NSG mice, and 3D structure of tibia (**G**), sagittal plane of trabecular (**H**), 3D structures of trabecular (**I**), transverse plane of trabecular (**J**), BV/TV (**K**) and BS/TV (**L**) were analyzed by micro-CT (*n* = 10)

To further confirm the above results, we used 5TGM1 and patient derived xenograft (PDX) models to observe the bone lesions mediated by LILRB4. Human *LILRB4* was expressed in 5TGM1 cells (Fig. 3A), and 5TGM1-vector and -*LILRB4* cells were injected into C57BL/6-*Rag2*^−/−^ mice [56]. Bioluminescence imaging showed that there was no significant difference between 5TGM1-vector and -*LILRB4* groups (Supplementary Fig. S7A, B), indicating that LILRB4 did not promote the cell proliferation. The bone damage of tibia was obvious in 5TGM1-*LILRB4* group (Fig. 3B), and trabecular bone damage was more profound (Fig. 3C, D), and BV/TV, BS/TV were lower in 5TGM1-*LILRB4* group (Fig. 3E, F). Histological staining showed that the cortical and trabecular bone was more intact in 5TGM1-vector group than that in -*LILRB4* group (Supplementary Fig. S7C). Next, fresh bone marrow samples of multiple myeloma were collected. CD45^−^/CD38^+^/LILRB4^+^ and CD45^−^/CD38^+^/LILRB4^−^ myeloma cells from the same patient were sorted, and the same number of cells were injected into NSG mice by intratibial injection, respectively. When mice died naturally, the bone lesion was scanned by micro-CT. CD45^−^/CD38^+^/LILRB4^+^ cells significantly promoted the bone damage (Fig. 3G), and trabecular bone damage was more serious in CD45^−^/CD38^+^/LILRB4^+^ group than that in CD45^−^/CD38^+^/LILRB4^−^ group (Fig. 3H-L). Collectively, ectopic LILRB4 significantly promoted bone lesion of multiple myeloma.

### RELT is upregulated in *LILRB4*-WT cells of multiple myeloma cell lines

**Fig. 4:**
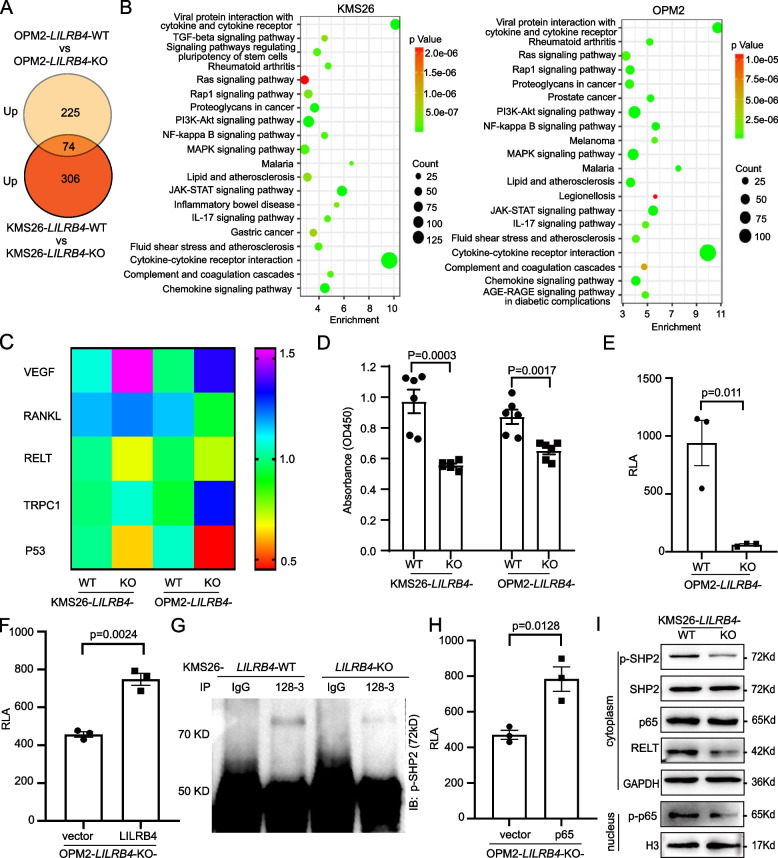
LILRB4 regulated the secretion of RELT by NF-κB signal pathway. The up-regulated cytokines in *LILRB4*-WT group of OPM2 and KMS26 cells (**A**). The enriched signal pathway of up-regulated cytokines (**B**). The real-time PCR results of *VEGF*, *RANKL*, *RELT*, *TRPC1* and *P53* (*n* = 3, **C**). The RELT level in conditioned medium of KMS26 and OPM2 (*n* = 6, **D**). Dual luciferase reporting system containing *RELT* promoter was transfected into OPM2-*LILRB4*-WT and -KO cells (*n* = 3, **E**). Dual luciferase reporting system and LILRB4 plasmid were transfected into OPM2-*LILRB4*-KO cells (*n* = 3, **F**). P-SHP2 was pull down by anti-LILRB4 (Clone No:128–3) (**G**). Dual luciferase reporting system and p65 plasmid were transfected into OPM2-*LILRB4*-KO cells (*n* = 3, **H**). P-SHP2, SHP2, p-p65, p65 and RELT were detected by western blotting (**I**)

To clarify the mechanism by which LILRB4 promotes bone lesion of multiple myeloma, we used cytokine arrays to identify the differentially expressed cytokines in the CM from *LILRB4*-WT and -KO cells. Compared with *LILRB4*-KO group, 380 and 299 cytokines were upregulated in KMS26 and OPM2-*LILRB4*-WT cells, respectively. The upregulated cytokines were enriched in multiple pathways including Ras, PI3K-Akt, NF-κB, MAPK, JAK-STAT, IL-17 and cytokine-cytokine receptor interaction (Fig. 4A, B). VEGF, RANKL, RELT, TRPC1 and P53 are related with osteolysis [57]. Real-time PCR results indicated that *RELT* and *P53* were significantly lower in *LILRB4*-KO than that in *LILRB4*-WT cells of KMS26 and OPM2 (Fig. 4C). Meanwhile, RNA-seq was used to screen the differentially expressed genes, and a total of 5496 differential genes were found, in which 835 genes were co-upregulated in *LILRB4*-WT cells (Supplementary Fig. S8A). These co-upregulated genes were enriched in the following pathways including cytokine-cytokine receptor interaction, chemokine signaling pathway, JAK-STAT and NF-κB signal pathway (Supplementary Fig. S8B), and RELT, IL27RA, IL4R, TGFB1 and TNFRSF1B were significantly higher in *LILRB4*-WT group than that in *LILRB4*-KO group (Supplementary Fig. S8C, D).

Based on the above results, we speculated that LILRB4 could promote the secretion of RELT. We detected the RELT level in CM from KMS26 and OPM2, and found that RELT was higher in *LILRB4*-WT group than that in *LILRB4*-KO group (Fig. 4D). Next, we constructed the dual luciferase reporter system with RELT promoter, and this system was transfected into the *LILRB4*-WT and -KO cells. The relative luciferase activity (RLA) could hardly be detected in *LILRB4*-KO cells, but the RLA was higher in *LILRB4*-WT cells (Fig. 4E). Meanwhile, LILRB4 plasmid and luciferase reporter system were co-transfected into OPM2-*LILRB4*-KO cells, and the co-transfection of *LILRB4* could also promote the activity of dual luciferase reporter system (Fig. 4F). We have previously reported that LILRB4 activated the NF-κB signal pathway by recruiting p-SHP2 [31, 32]. Next, we confirmed that LILRB4 could recruit p-SHP2 in multiple myeloma cells by Co-IP (Fig. 4G), and the co-transfection of p65 could significantly promote the activity of dual luciferase reporter system (Fig. 4H). LILRB4 and p65 could both promote the expression of RELT. Western blotting showed that, compared to *LILRB4*-KO group, p-SHP2 and RELT were significantly higher in cytoplasm, and p-p65 was also significantly elevated in the nucleus in *LILRB4*-WT group (Fig. 4I and Supplementary Fig. S9A).

Taking together, these studies indicated that LILRB4 could recruit p-SHP2 to active NF-κB signal pathway to promote the secretion of RELT.

### RELT induced osteoclastogenesis and bone lesions in MM models

**Fig. 5:**
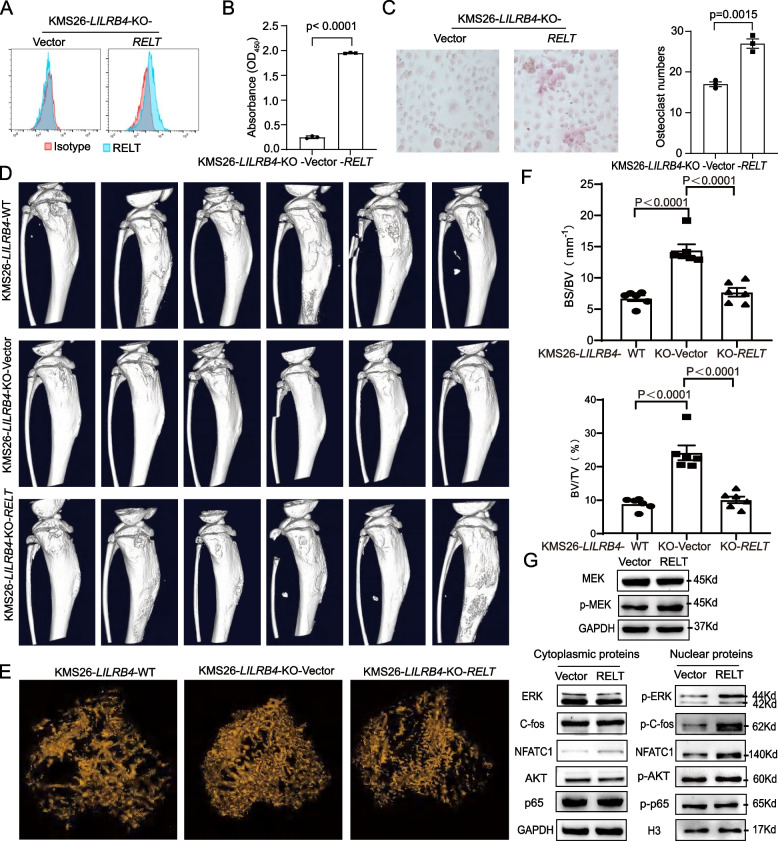
RELT regenerated bone damage in *LILRB4*-KO cells. *RELT* was overexpressed in KMS26-*LILRB4*-KO cells (**A**), and the content of RELT in conditioned medium was determined by ELISA (*n* = 3, **B**). The conditioned medium from KMS26-*LILRB4*-KO-vector or -*RELT* was used to treat primary bone marrow monocytes (*n* = 3, **C**). 3D structure of tibia (**D**), 3D trabecular (**E**), BV/TV and BS/TV (**F**) were analyzed by micro-CT (*n* = 18). RELT signal pathway was detected by western blotting (**G**)

Whether RELT promotes osteolytic lesions in multiple myeloma has not been reported. *RELT* was overexpressed in *LILRB4*-KO cells (named *LILRB4*-KO-*RELT*), and the RELT level in the CM from *LILRB4*-KO-*RELT* cells was significantly higher than that in *LILRB4*-KO-vector cells (Fig. 5A, B). Next, the CM from *LILRB4*-KO-*RELT* or -vector cells were used to treat primary bone marrow monocytes, and the CM of *LILRB4*-KO-*RELT* cells significantly promoted the osteoclastogenesis (Fig. 5C). Meanwhile, the similar phenotype was observed, that is, the recombinant human RELT could induce the differentiation and maturation of osteoclasts (Supplementary Fig. S9B). Next, KMS26-*LILRB4*-WT, KMS26-*LILRB4*-KO-*RELT* and -vector cells were injected into NSG mice by intratibial injection. After 7 days, mice were sacrificed under anesthesia, and micro-CT was used to scan the bone lesion. 3D structure of tibia showed that the surface of bone cortex was uneven, and multiple obvious bone injures were observed, and this phenotype was more severe in *LILRB4*-WT group as well as *LILRB4*-KO-*RELT* group (Fig. 5D). 3D-trabecular showed that trabecular loss was more severe, BV/TV and BS/TV was lower in *LILRB4*-WT and *LILRB4*-KO-*RELT* group (Fig. 5E-F). These results indicated that *RELT* overexpression in *LILRB4*-KO cells could completely restore the bone damage caused by LILRB4, then RELT does play an important role in sustaining the LILRB4 osteolytic function.

Next, we collected bone marrow samples from 5 cases of multiple myeloma, and found that higher level of LILRB4 in CD45^−^/CD38^+^ multiple myeloma cells seems to correspond to a tendency towards more RELT level in serum (Supplementary Fig. S9C, D). RELT could promote the proliferation of T cells [58], however, it is unknown how RELT regulates osteoclastogenesis. The CM of KMS26-*LILRB4*-KO-vector or -*RELT* cells was used to treat Raw264.7 cells. After 4 days, the cells were lysed, and cytoplasmic and nuclear proteins were extracted, respectively. In the CM-RELT-treated group, the level of p-MEK was significantly increased in cytoplasm, and p-ERK, p–c-fos and NFATC1 were also significantly elevated in the nucleus, but p-AKT and p-p65 were no significant changed (Fig. 5G and Supplementary Fig. S9E-J). p–c-fos and NFATC1 are markers of osteoclast maturation and differentiation. Therefore, RELT promoted the proliferation, maturation and differentiation of osteoclast by p-MEK/p-ERK/p–c-fos and NFATC1 signal pathway.

### The knockout of *LILRB4*, anti-LILRB4 alone or in combination with BTZ could alleviate bone lesions

**Fig. 6:**
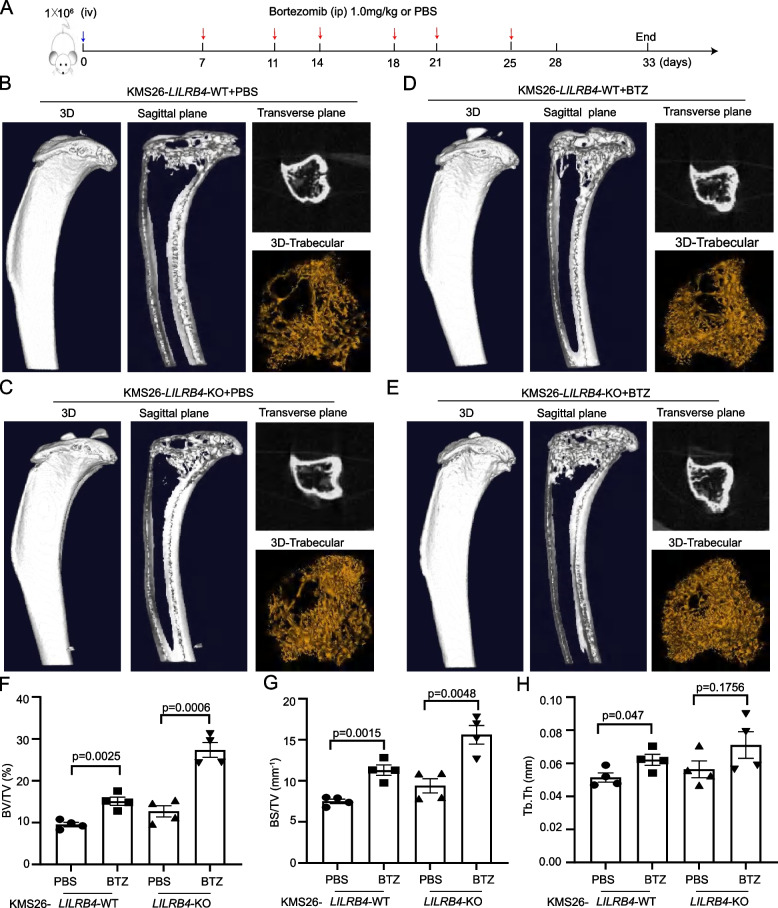
BTZ was used to treat *LILRB4*-WT and -KO mouse models. The schematic of BTZ treated KMS26 xenograft model (*n* = 16, **A**). 3D structure of tibia, sagittal and transverse plane of trabecular and 3D trabecular in different group were shown (**B-E**). BV/TV, BS/TV and Tb.Th (trabecular thickness) were determined by micro-CT (*n* = 16, **F–H**)

Bortezomib (BTZ) is a protease inhibitor, a first-line therapy for the treatment of multiple osteoma in the clinical [59]. Little is known whether BTZ can inhibit bone damage in multiple myeloma with the depletion of *LILRB4*. KMS26-*LILRB4*-WT or -KO cells were injected into NSG mice by tail vein, and BTZ or PBS was used to treat xenograft models twice a week (Fig. 6A). After 33 days, all mice were sacrificed under anesthesia, and bone lesion was canned by micro-CT. Compared with *LILRB4*-KO group, *LILRB4*-WT cells significantly promoted the bone injury (Fig. 6B, C). After BTZ treatment, the destruction of trabecular was significantly reversed, especially in *LILRB4*-KO group (Fig. 6D, E), and BV/TV, BS/TV were significantly elevated (Fig. 6F-G). Tb.Th was also increased, although not significantly (Fig. 6H).

**Fig. 7:**
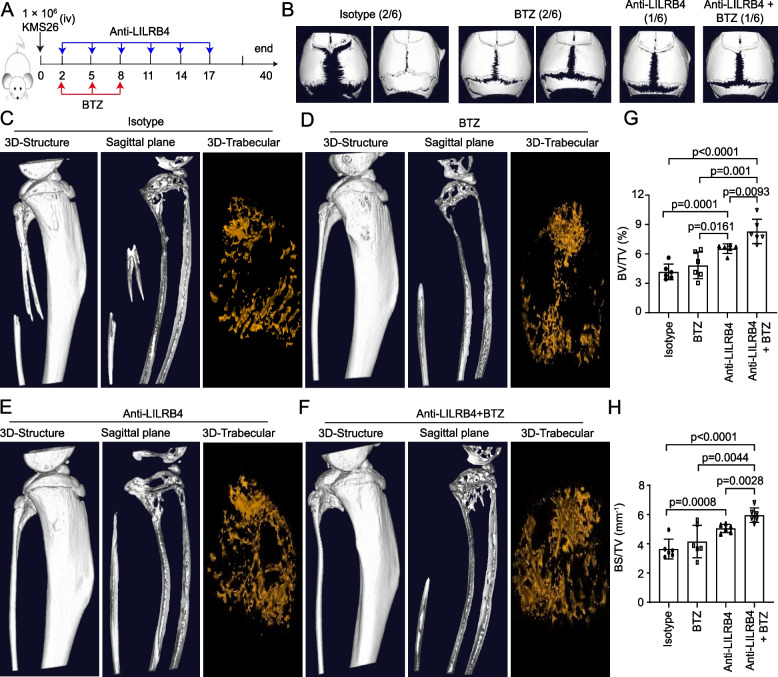
Anti-LILRB4 alone or in combination with BTZ treated KMS26 mouse model. The schematic to show the treatment of anti-LILRB4 and BTZ (**A**). The crack of cranial sutures was scanned by micro-CT (**B**). The 3D-structure, sagittal plane and 3D-trabecular in tibia were scanned by micro-CT (*n* = 24, **C-F**), and BV/TV, BS/TV were analyzed (**G**, **H**)

Next, we used anti-LILRB4 and BTZ to treat KMS26 mouse model (Fig. 7A). After 40 days of tumor injection, 3 out of 6 mice (50%) died in isotype and only BTZ treated groups, 1 mouse (16.67%) died in anti-LILRB4-treated group, and no mice died in the combination therapy group (anti-LILRB4 + BTZ). In isotype or BTZ treated groups, 2 out of 6 mice (30%) showed significant cracking in their cranial sutures, and only 1 mouse showed cracking in anti-LILRB4 and combination therapy (Fig. 7B). The bone erosion, bone fracture and trabecular bone loss were more severe in isotype or BTZ treated groups than that in anti-LILRB4 or combination therapy groups (Fig. 7C-F). BV/TV and BS/TV were significantly increased in anti-LILRB4 and combination therapy groups (Fig. 7G, H).

The above results indicated that anti-LILRB4 alone or in combination with BTZ have the potential to inhibit bone damage in multiple myeloma.

## Discussion

LILRB4 is a molecular marker of M4 and M5 acute myeloid leukemia (AML), which mediates the immune escape and extramedullary infiltrates of AML [31, 32]. LILRB4 is also expressed on the surface of tumor related macrophages, MDSC and other immune cells in the tumor microenvironment, forming a suppressive tumor microenvironment to promote tumor cell immune escape, and is a potential target for immunotherapy [35, 38, 60]. In addition, LILRB4 has been reported to be expressed in non-small cell lung cancer [42], therefore, elucidating the expression pattern of LILRB4 in tumor cells is fundamental for investigating the role of LILRB4. The surface proteomic analysis of multiple myeloma identified LILRB4 as a potential immunotherapeutic target [61]. In this study, we first focused on the expression of LILRB4 in various tumor tissue arrays. Unexpectedly, LILRB4 was expressed in 50% of multiple myeloma samples, and flow cytometry further validated this result. Although LILRB4 was also expressed in several other solid tumors, the level was lower, therefore, we focused on and explored the relationship between LILRB4 and multiple myeloma.

Multiple myeloma is typically characterized by multiple bone lesion. We analyzed the relationship between patient imaging data and LILRB4 expression, and found that the patients with high expression level of LILRB4 had more severe bone damage. *In vitro* data also showed that LILRB4 could promote the differentiation and maturation of osteoclasts, but had no significant effect on the differentiation and maturation of osteoblasts. Next, we used differential animal models to validate this result. In xenograft model, the knockout of *LILRB4* in KMS26 and OPM2 could significantly inhibit the destruction of bone cortex and trabecular. In syngeneic model, 5TGM1 cells were injected into the C57BL/6-*Rag2*^−/−^ mice by tail vein. The overexpression of *LILRB4* in 5TGM1 cells significantly promoted bone damage. A paper reported that LILRB4 mediated the migration of MM cell line (NCI-H929) [62]. However, we did not observe any effect of LILRB4 on MM cell migration in differential animal models. Finally, PDX models also showed that CD45^−^/CD38^+^/LILRB4^+^ cells had stronger bone destruction.

**Fig. 8:**
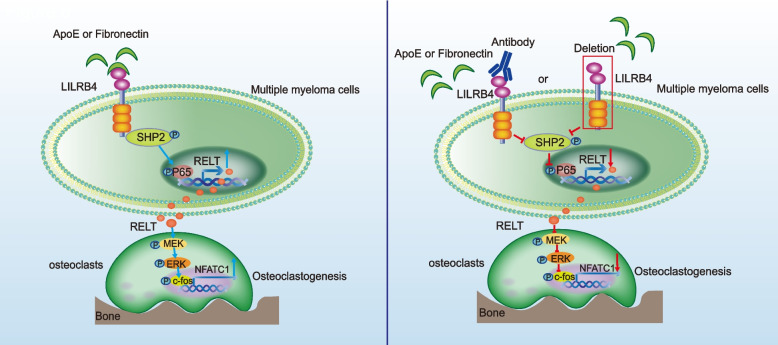
Schematic depicting the activation and blockade of the LILRB4 signaling pathway to regulate bone lesion in multiple myeloma. ApoE or fibronectin bound to LILRB4 to recruit p-SHP2, then RELT was secreted in multiple myeloma. Next, RELT could active p-MEK/p-ERK/p–c-fos and NFATC1 signal pathway to promote the proliferation of osteoclasts. Bone lesions could be alleviated by knockout of *LILRB4* or anti-LILRB4 to blocking the LILRB4 signaling pathway

LILRB4 breaks the balance between osteoclasts and osteoblasts, resulting in more severe bone damage in the multiple myeloma mouse model. Subsequently, we explored the mechanism of LILRB4 promoting bone injury in multiple myeloma. The results of cytokine microarray, RNA-Seq and real time-PCR showed that RELT was the most significant upregulated cytokine in *LILRB4*-WT group, and dual luciferase reporting system showed that LILRB4 could regulate the secretion of RELT by p-SHP2-NF-κB signal pathway (Fig. 8). RELT, a new member of the tumor necrosis factor receptor superfamily, is selectively expressed in hematopoietic tissues and activates transcription factor NF-κB [58], and negatively regulates the activity of T cells [63]. Serum RELT (soluble) was elevated in patients with gastric cancer and breast cancer [64, 65], especially in B-cell lymphomas, but RELT is an orphan receptor, and its ligand is unknown [66]. How RELT promotes the differentiation and maturation of osteoclasts has not been reported. RELT binds to its putative receptor(s) on osteoclasts, and drivers the maturation of osteoclasts. In this study, our data confirmed that RELT promoted the proliferation of osteoclasts by p-MEK/p-ERK/p–c-fos and NFATC1 signal pathway (Fig. 8).

Finally, we tried to explore the role of LILRB4 in treatment of multiple myeloma. The depletion of *LILRB4* or anti-LILRB4 alone or in combination with BTZ could delay the progression of multiple myeloma, and significantly inhibit the bone damage (Fig. 8). LILRB4 did not affect the proliferation and metastasis of multiple myeloma, but LILRB4 blocking antibody could prevent the binding of LILRB4 to its ligands, therefore, anti-LILRB4 could block its signal pathway to inhibit the RELT secretion, then alleviate bone damage. Our data supported that LILRB4 signal pathway promoted the differentiation and mature of osteoclasts by secreting RELT, and LILRB4 had the potential to be a new target for the treatment of multiple myeloma.

### Supplementary Information

Supplementary Material 1.

## Data Availability

The data generated in this study are available from the corresponding author upon reasonable request.

## Abbreviations

LILRB4: Leukocyte Ig-like receptor B family 4
BMSCs: Bone marrow stromal cells
IL-6: Interleukin-6
OPG: Osteoprotegerin
ASCT: Autologous stem cell transplant
APC: Antigen presenting cells
Ts: T suppressor cells
AML: Acute myeloid leukemia
MDSCs: Myeloid-derived suppressor cells
CAR-T: Chimeric antigen receptor T
ADC: Antibody drug conjugates
CLL: Chronic lymphocytic leukemia
BTZ: Bortezomib
DMEM: Dulbecco’s modified Eagle’s medium
NSG: NOD-SCID IL2Rγ-null
CM: Conditioned medium
CDS: Coding sequence
TCGA: The Cancer Genome Atlas
BRCA: Breast invasive carcinoma
CHOL: Cholangiocarcinoma
ESCA: Esophageal carcinoma
HNSC: Head and Neck squamous cell carcinoma
KICH: Kidney chromophobe
KIRC: Kidney renal clear cell carcinoma
KIRP: Kidney renal papillary cell carcinoma
PRAD: Prostate adenocarcinoma
SKCM: Skin Cutaneous Melanoma
STAD: Stomach adenocarcinoma
UCEC: Uterine Corpus Endometrial Carcinoma
IHC: Immunohistochemistry
RLA: Relative luciferase activity
