# Supplementary material for: LILRB4 on multiple myeloma cells promotes bone lesion by p-SHP2/NF-κB/RELT signal pathway
Source: J Exp Clin Cancer Res. 2024 Jul 1;43:183. doi: 10.1186/s13046-024-03110-y (PMC11218313; doi:10.1186/s13046-024-03110-y)
Supplement: Supplementary file 1 — Supplementary Material 1. [file 13046_2024_3110_MOESM1_ESM.docx]

**Supplementary Table. S1 The information of patients with multiple myeloma**

| Clinical characteristics | | | LILRB4 levels | | p value |
| --- | --- | --- | --- | --- | --- |
|  |  |  | ≥10% | <10% |  |
| Sex | F | 11 (68.75%) | 6 | 5 | 0.9999 |
|  | M | 5 (31.25%) | 3 | 2 |  |
| Age | <65 | 10 (62.5%) | 5 | 5 | 0.3069 |
|  | ≥65 | 6 (37.5%) | 5 | 1 |  |
| Stage | Ⅱ | 3 (18.75%) | 0 | 3 | 0.0625 |
|  | Ⅲ | 13 (81.25%) | 9 | 4 |  |
| Renal impairment | Yes | 3 (18.75%) | 1 | 2 | 0.5500 |
|  | No | 13 (81.25%) | 8 | 5 |  |
| Type of myeloma | IgG | 7 (43.75%) | 3 | 4 | 0.6145 |
|  | Non-IgG | 9 (56.25%) | 6 | 3 |  |
| Extramedullary disease | Yes | 11 (68.75%) | 9 | 2 | 0.0048 |
|  | No | 5 (31.25%) | 0 | 5 |  |

**Supplementary Figures**


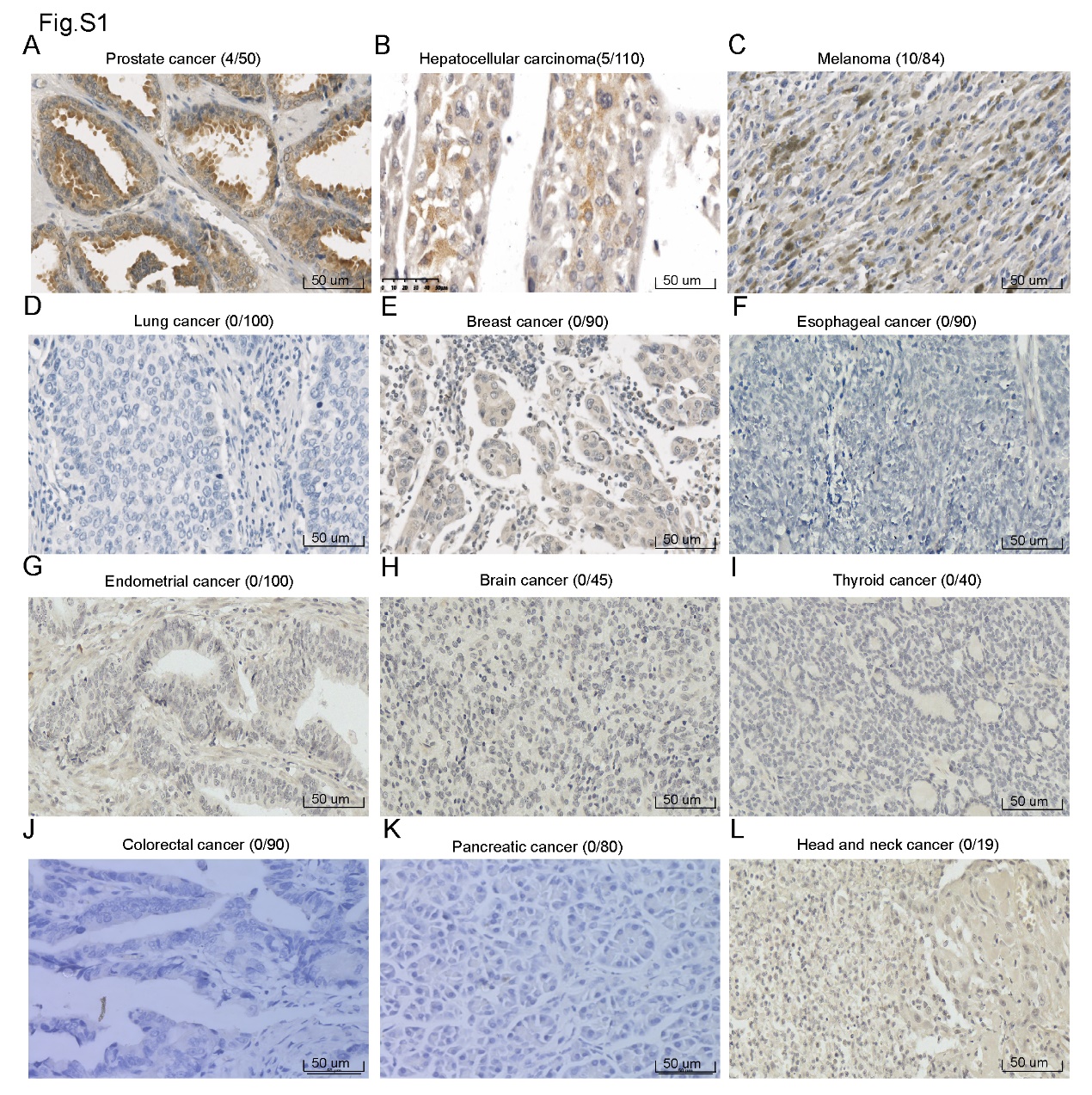


**Supplementary Figure. S1 LILRB4 was detected by IHC in different tumor tissue arrays.**

LILRB4 was detected by immunohistochemistry in various tumor tissue arrays including prostate cancer (A), hepatocellular carcinoma (B), melanoma (C), lung cancer (D), breast cancer (E), esophageal cancer (F), endometrial cancer (G), brain cancer (H), thyroid cancer (I), colorectal cancer (J), pancreatic cancer (K) and head and neck cancer (L). The antibody targeting LILRB4 is Rab 128-3, and the dilution is 1:200.


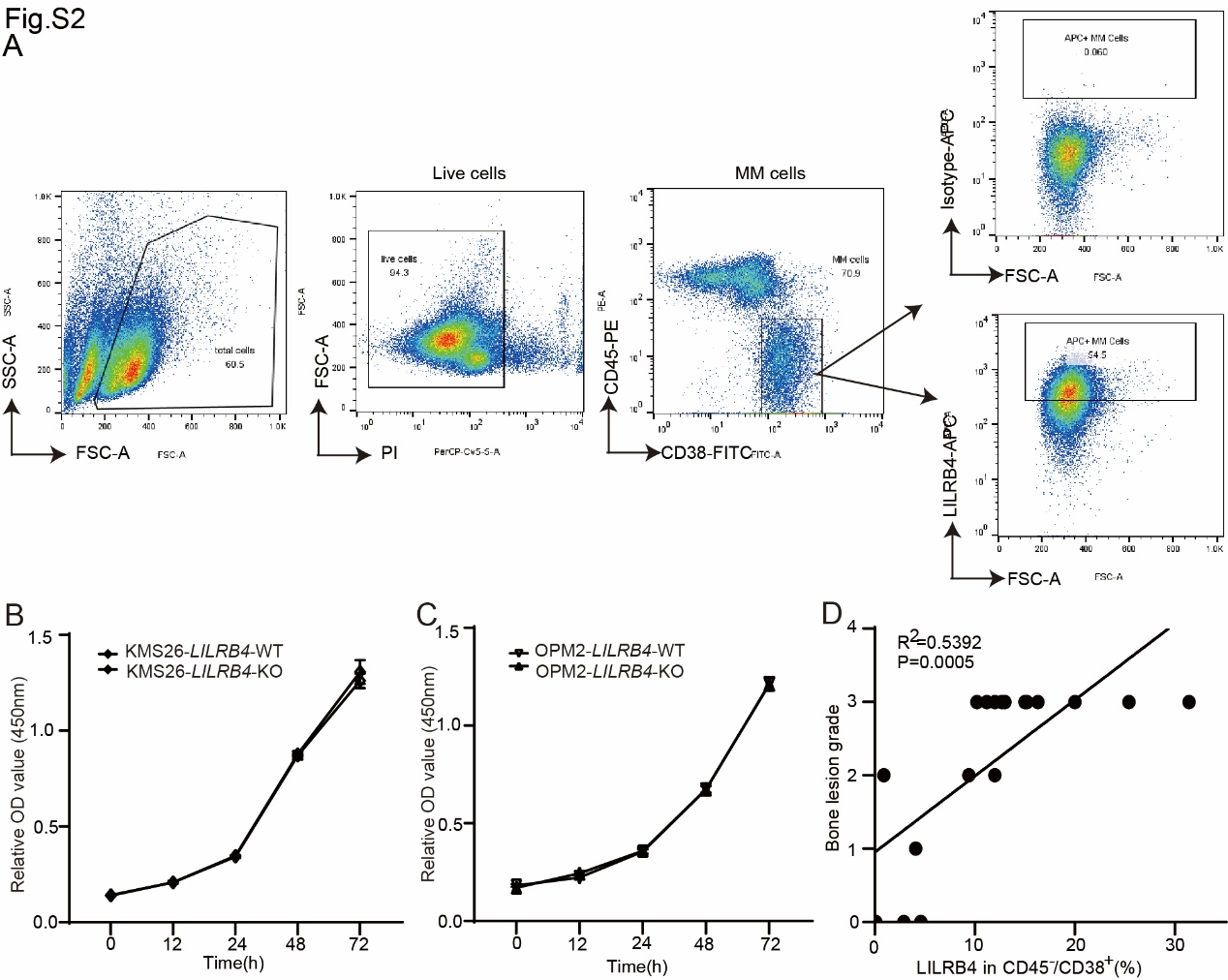


**Supplementary Figure. S2 The effect of LILRB4 on proliferation and bone damage in multiple myeloma.**

LILRB4 was detected by flow cytometry in fresh patient samples and the gating strategy was shown (n=9, **A**). The proliferation was assessed by a CCK-8 kit. The difference in proliferation rate was not observed in KMS26-*LILRB4*-WT and –KO and OPM2-*LILRB4*-WT, -KO cells (B, C). Data represent the mean ± SEM (n = 3). The correlation trend between the LILRB4 level and the patient's bone injury (n=16, D).


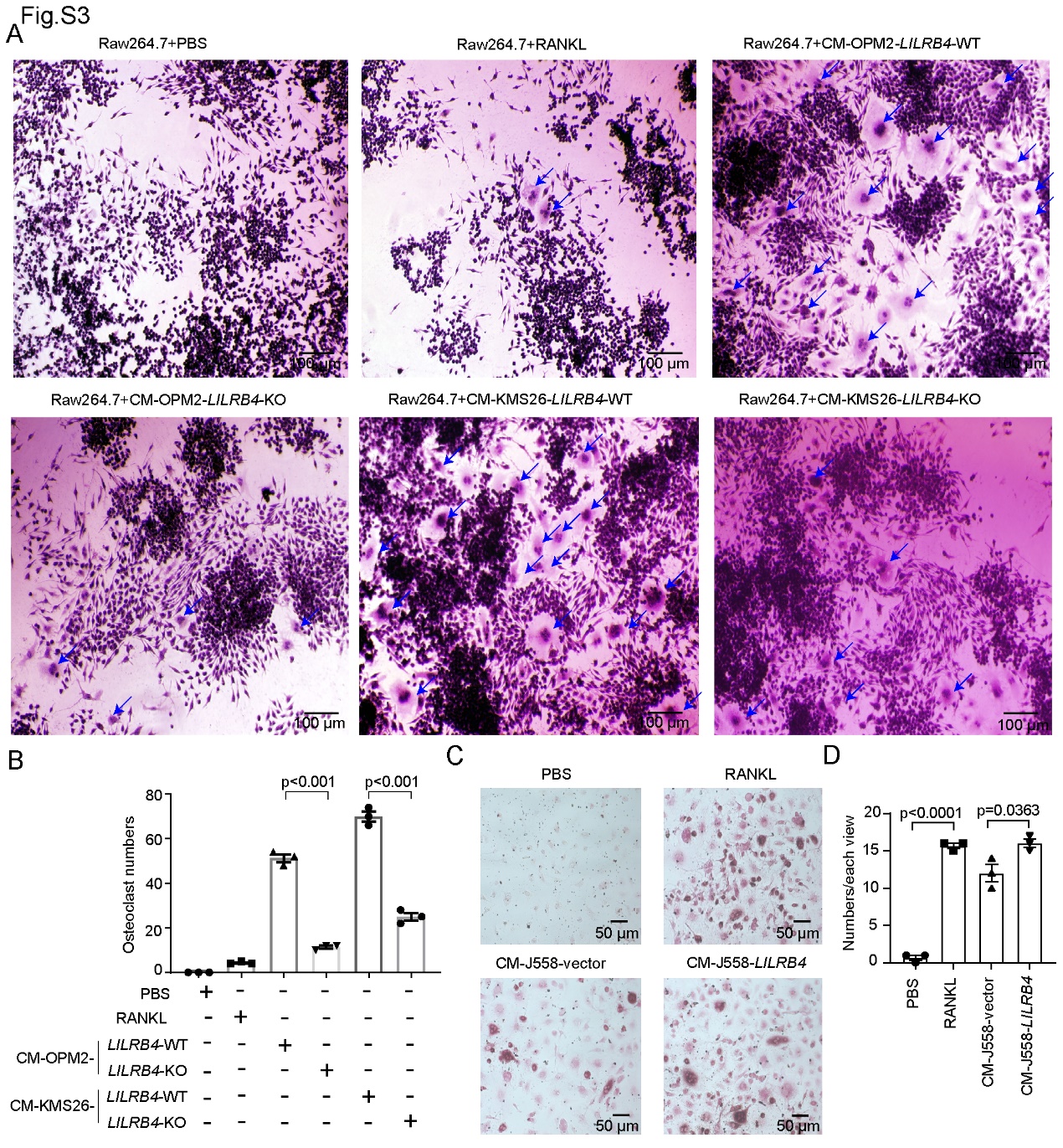


**Supplementary Figure. S3 The conditioned medium of LILRB4 induced osteoclastogenesis *in vitro*.**

Raw264.7 cells were cultured with conditioned medium (CM) from *LILRB4*-WT and -KO cells in the presence of M-CSF (25 ng/ml) and RANKL (100 ng/ml) for 4 days. Osteoclasts were stained for TRAP staining, and the cells with more than 3 nuclei (indicated by blue arrow) were osteoclasts (**A**). The number of osteoclasts in all fields were counted, and data represent the mean ± SEM (n = 3, **B**). Bone marrow derived mononuclear was cultured with CM from J558-vector or -*LILRB4* in the presence of M-CSF (25 ng/ml) and RANKL (100 ng/ml) for 6 days (**C**), and data represent the mean ± SEM (n = 3, **D**).


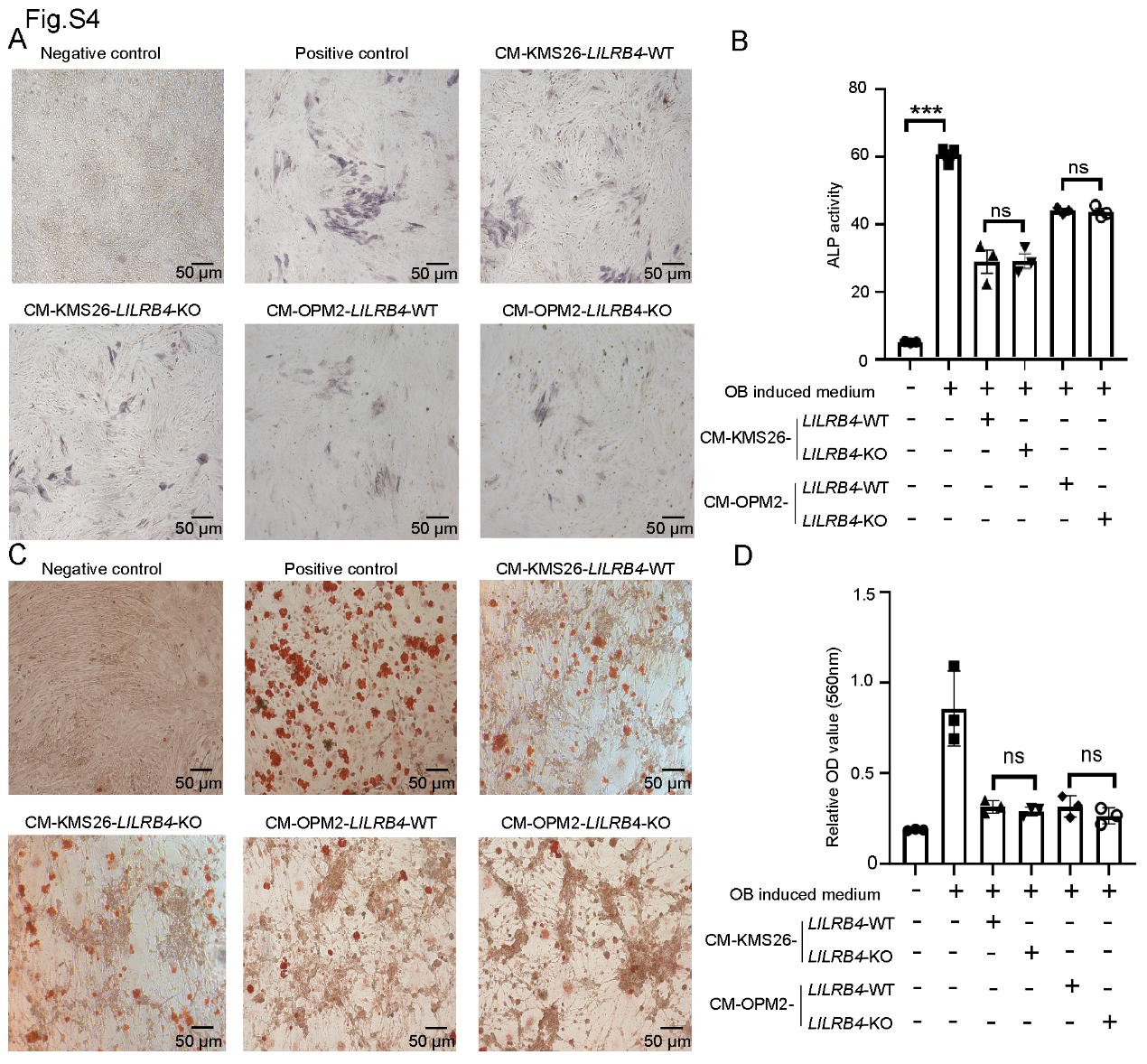


**Supplementary Figure. S4 LILRB4 did not promote the differentiation and maturation of osteoblasts.**

MC3T3-E1 was treated with CM from *LILRB4*-WT or -KO cells in the presence of induced medium. The activity of ALP was assayed by staining (A) and ELISA (B). The calcium nodules were stained by alizarin red (C), and were quantitatively analyzed (D). Data represent the mean ± SEM (n = 3). ***p<0.001, ns means no significant.


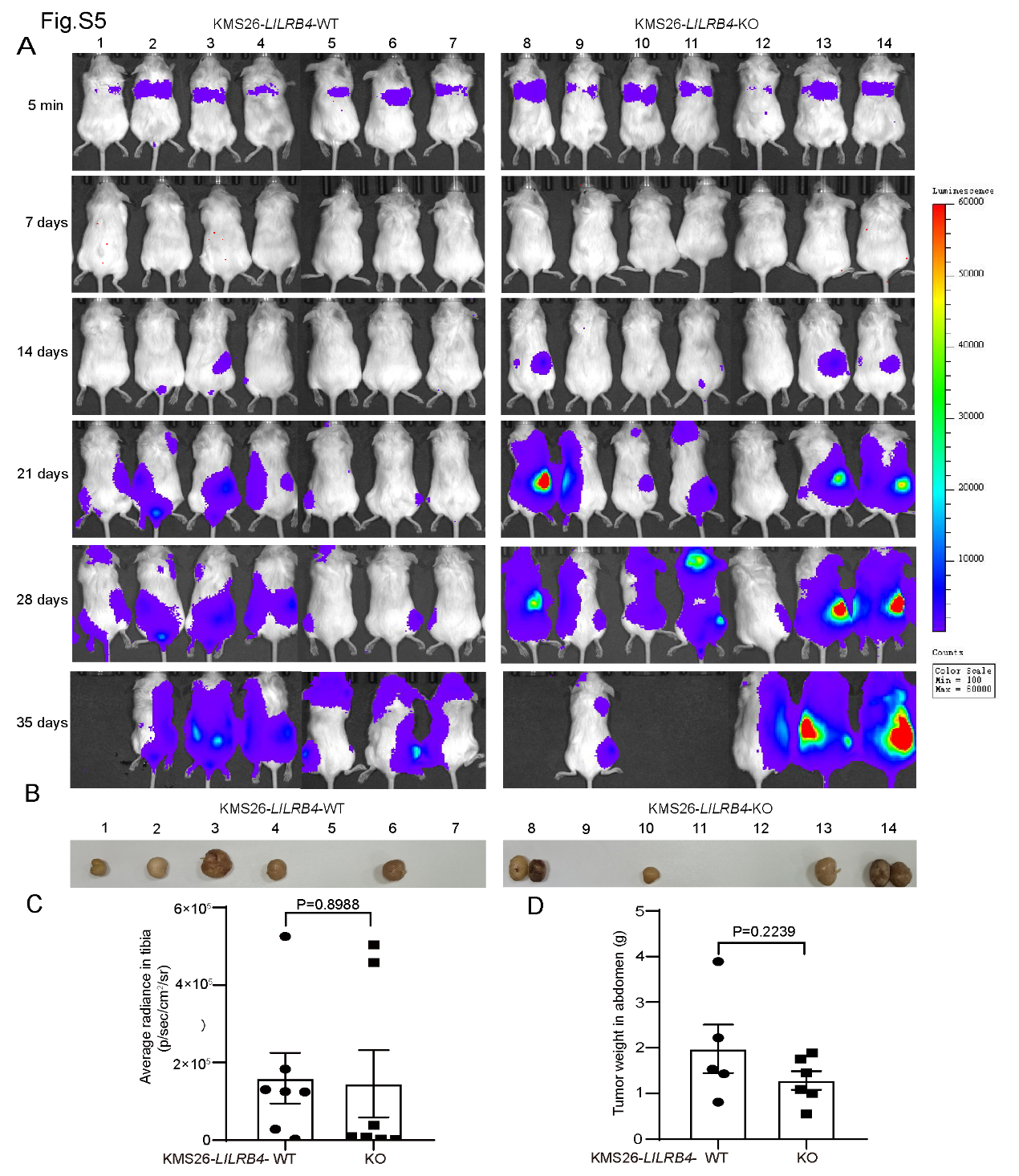


**Supplementary Figure. S5 LILRB4 does not affect proliferation and metastasis in the KMS26 xenograft model.**

Luminescence imaging was used to monitor the progression of tumors at 0, 7, 14, 21, 28 and 35 days (n=14) (A). 5 mice developed 5 abdominal tumors in *LILRB4*-WT group, and 4 mice showed 6 abdominal tumors in *LILRB4*-KO group (B). Quantitative analysis of luminescence intensity of the tibia(n=14) (C). Analyze the weight of abdominal tumors(n=11) (D).


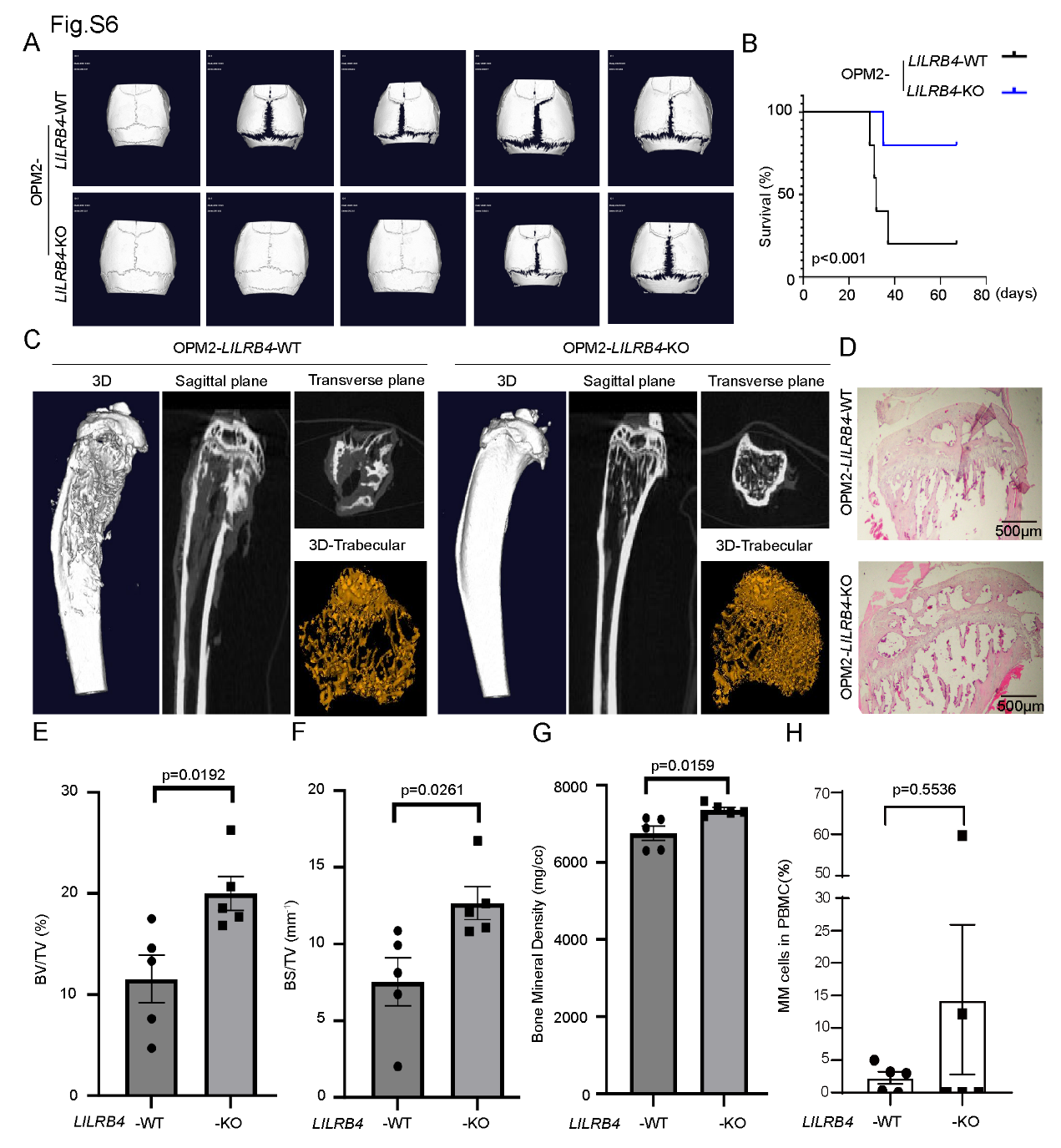


**Supplementary Figure. S6 LILRB4 promoted the bone damage in OPM2 xenograft model.**

OPM2-*LILRB4*-WT and -KO cells were injected into NSG mice, and micro-CT showed the cracking of the bone suture of the skull (A), and the survival curve was plot (B). The 3D structure of tibia, sagittal, transverse and corona plane of trabecular and 3D-trabecular were showed (C). Bone lesions were observed in the H&E staining (D).BV/TV (E), BS/TV (F) and bone mineral density (G) were analyzed by micro-CT (n=10). OPM2 cells was detected from peripheral blood (n=10) (H).


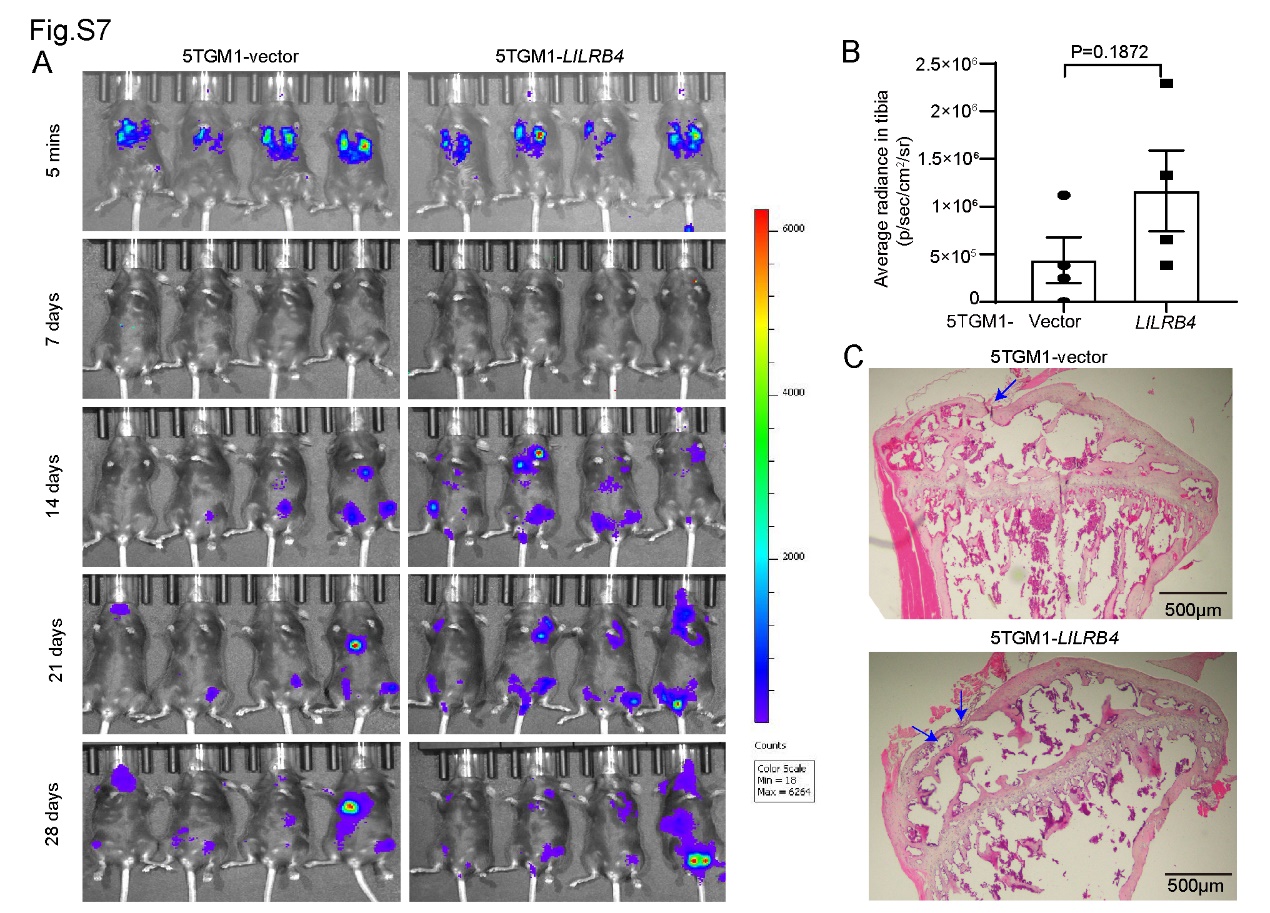


**Supplementary Figure. S7** **LILRB promotes the progression of 5TGM1 by luminescence imaging.**

1×10^6^ 5TGM1-vector and -*LILRB4* cells were injected into C57BL/6-*Rag2*^-/-^ mice by iv, and the progression was monitored by luminescence imaging on 0, 7, 14, 21 and 28 days (n=8) (A). Quantitative analysis of luminescence intensity of the tibia(n=8) (B). Bone lesions were observed in H&E staining (blue arrows indicate cortical bone lesions) (C).


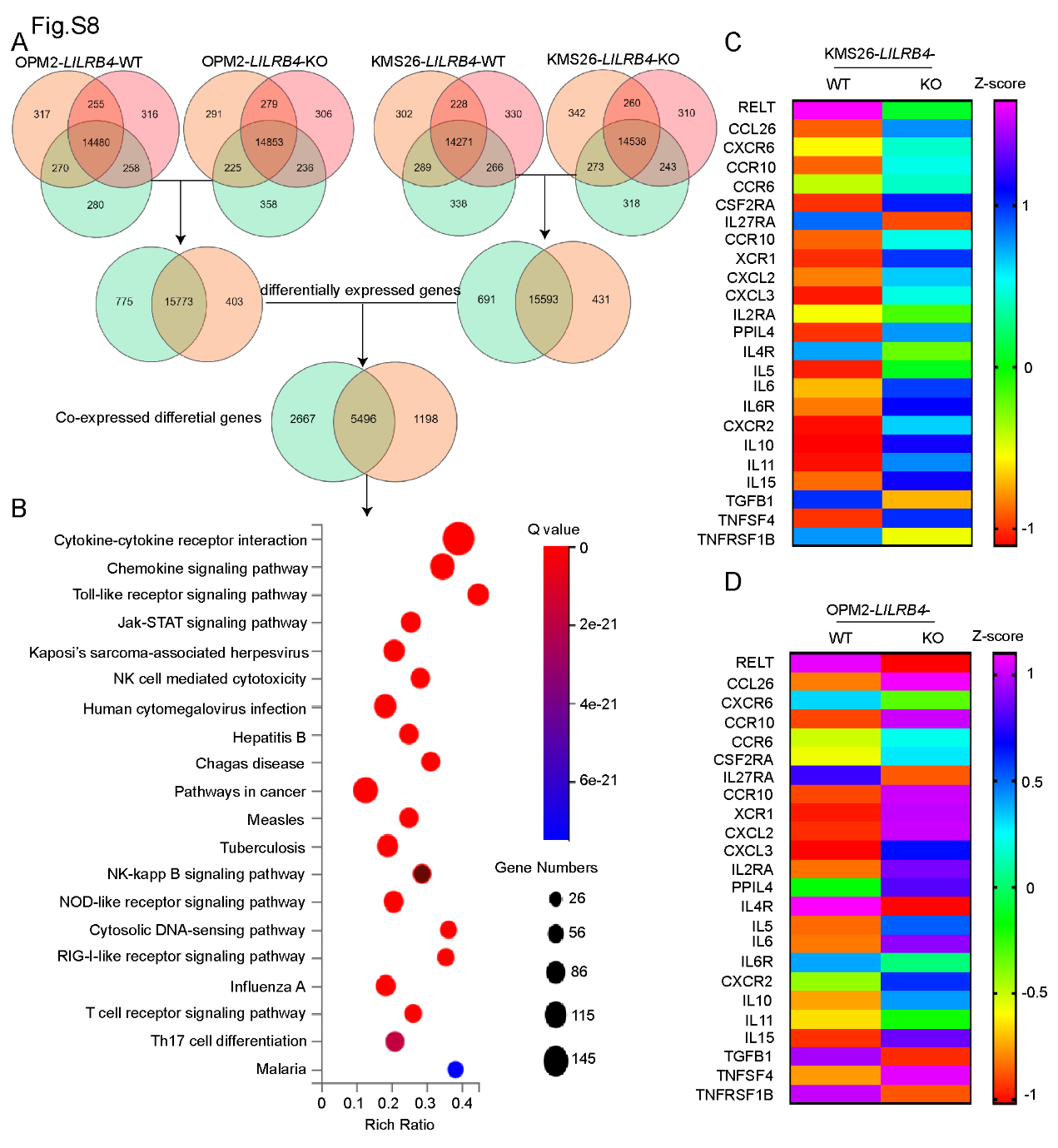


**Supplementary Figure. S8 RNA-seq was used to identify differentially expressed cytokines.**

The differentially expressed genes were identified in *LILRB4*-WT and -KO cells by RNA-seq (n=6, A), and the co-upregulated genes in *LILRB4*-WT group were enriched by GO (B). The levels of cytokines associated with bone injury in multiple myeloma in KMS26 (C) and OPM2 (D).


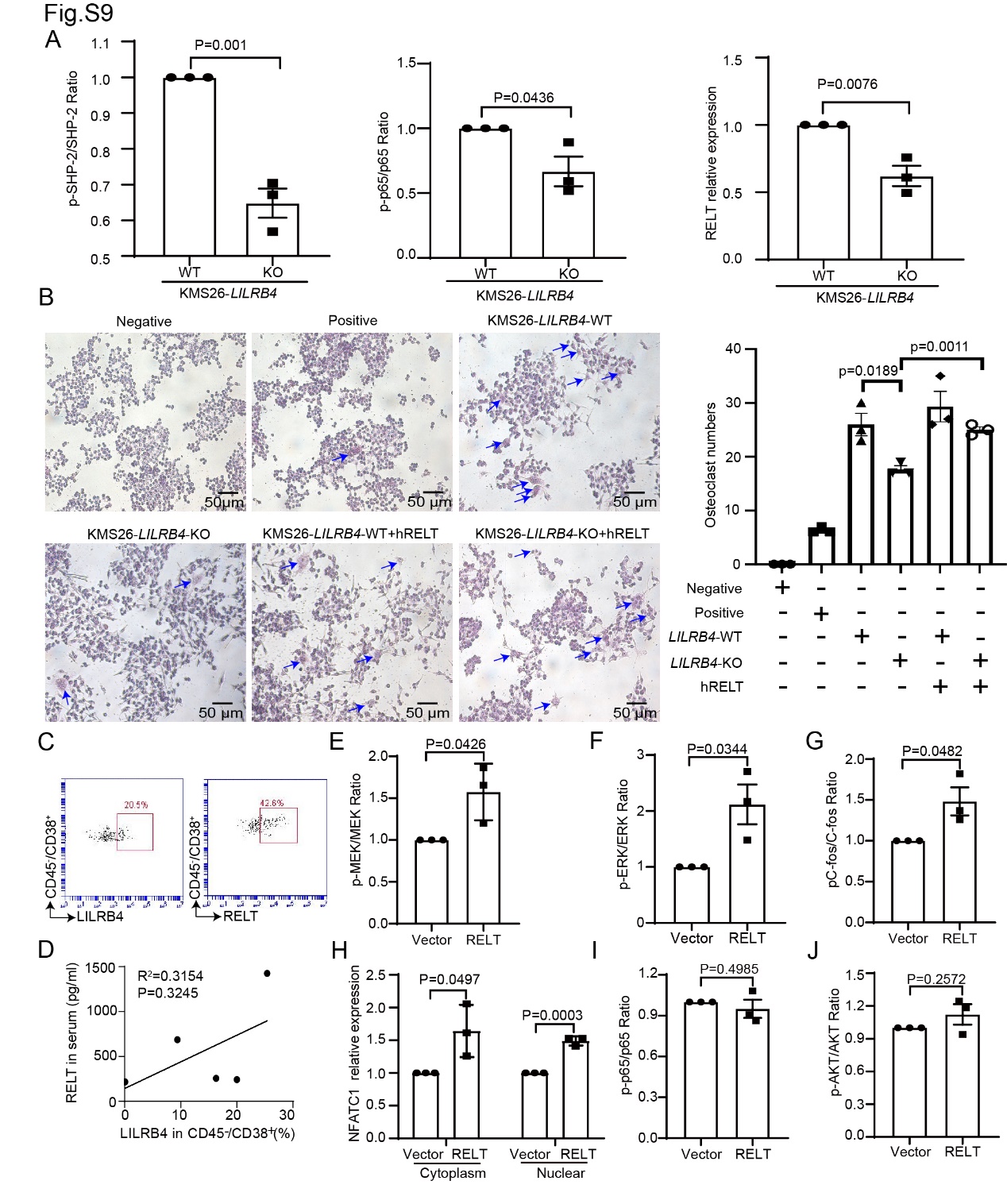


**Supplementary Figure. S9 The upregulated expression of RELT by LILRB4 can enhance osteoclast maturation and bone damage.**

SHP-2, p65 phosphorylation and RELT were quantified through image J (n=3, A). The conditioned medium of *LILRB4*-WT and -KO cells was used to treat Raw264.7 with or without exogenous RELT in the presence of the induced medium (Blue arrow indicated the osteoclasts, n=3, B). The levels of LILRB4 and RELT were detected in primary bone marrow samples in patients with multiple myeloma (n=5, C), and the correlation between LILRB4 and RELT level in serum was shown (n=5, D). P-MEK/ MEK (n=3, E), p-ERK/ ERK (n=3, F), p-C-fos/ C-fos (n=3, G), NFATC1 (n=3, H), p-p65/ p65 (n=3, I), p-AKT/AKT (n=3, J) were quantified using Image J (n=3, D).
